# A Novel Oligonucleotide Pair for Genotyping Members of the *Pseudomonas* Genus by Single-Round PCR Amplification of the *gyrB* Gene

**DOI:** 10.3390/mps1030024

**Published:** 2018-07-02

**Authors:** Betina Cecilia Agaras, Claudio Valverde

**Affiliations:** Laboratorio de Bioquímica, Microbiología e Interacciones Biológicas en el Suelo, Departamento de Ciencia y Tecnología, Universidad Nacional de Quilmes—CONICET, B1876BXD Bernal, Argentina; valverdecl@hotmail.com

**Keywords:** *Pseudomonas*, *gyrB*, MLSA, PCR-RFLP

## Abstract

*Pseudomonas* is a phylogenetically diverse bacterial genus which is broadly distributed in different ecological niches, and whose taxonomy is continuously under revision. For that purpose, *gyrB* is one of the housekeeping genes routinely used for multilocus sequence analysis (MLSA). As we noticed that there was not a single primer pair available in the literature suitable for direct sequencing of this gene, we decided to design a unique oligonucleotide pair and to set up a polymerase chain reaction (PCR) protocol to obtain a single amplicon for the entire *Pseudomonas* genus. Based on the available *gyrB* sequence from 148 *Pseudomonas* species, we identified highly conserved regions to design oligonucleotides without fully degenerate positions. We then set up cycling conditions for achieving high specificity and yield of the PCR protocol. Then, we showed that the amplicons produced with this procedure were appropriate for direct sequencing with both primers, obtaining more than 95% of amplicons coverage. Finally, we demonstrated that a PCR-RFLP (restriction fragment length polymorphism) approach served to differentiate among *Pseudomonas* species, and even between members of the same species.

## 1. Introduction

DNA gyrase is an essential bacterial enzyme that catalyzes the ATP-dependent negative super-coiling of circular double-stranded DNA (dsDNA) [1]. Genes encoding the two subunits of this type II topoisomerase, *gyrA* and *gyrB*, have been widely used as markers in phylogenetic studies of different prokaryotic taxa [2,3,4]. Also, the sequences of *gyrA* and *gyrB* alleles from quinolone-resistant isolates have been characterized to understand the evolutionary dynamics of antibiotic resistance, and to promote development of novel drugs [5,6]. In particular, *gyrB* has been considered a useful housekeeping gene for multilocus sequence analysis (MLSA) in several bacterial genera [7,8,9,10,11]. These studies typically used *gyrB* polymerase chain reaction (PCR) primers that were designed on the basis of the amino acid sequence of GyrB polypeptides from different bacterial species, and therefore, they contain several degenerate bases [2,12,13]. For this reason, some authors had to utilize different primer pair combinations to obtain *gyrB* amplicons for phylogenetic analyses of a whole bacterial genus, such as *Pseudomonas* [14,15]. Other authors designed specific primers for their particular purposes based on the *gyrB* nucleotide sequence of certain bacterial species [16,17,18,19].

*Pseudomonas* is a phylogenetically diverse bacterial genus of high interest in clinical, environmental, and molecular sciences. This is mainly due to the fact that *Pseudomonas* species are broadly distributed in different ecological niches, they can be isolated with relative ease in a variety of selective media, there are hundreds of fully sequenced genomes available, and they are easy to manipulate with genetic engineering tools [20,21]. Since pseudomonads can behave as human, plant or insect pathogens, as well as plant growth-promoting rhizobacteria or key players in bioremediation processes [22], molecular methods that allow a rapid identification of isolates belonging to this genus with a single PCR reaction are useful and desired tools [20,23]. We noticed that there was not a single primer pair in literature that could be employed for the amplification of the entire *Pseudomonas* genus, but only specific primers for some species or groups [16,17,24,25]. In this report, we present a novel primer pair and a PCR protocol for the amplification of a larger fragment of the *gyrB* gene from representative species of the entire *Pseudomonas* genus that is suitable for downstream applications such as restriction fragment length polymorphism (RFLP) analysis or direct sequencing for taxonomic purposes.

## 2. Materials and Methods

### 2.1. Bacterial Strains

Type strains and isolates used to set up the PCR protocol are listed in Table 1. All strains were cultured on nutrient agar (NA; 40 g/L tryptone soy agar; 5 g/L yeast extract; Biokar, Beauvais, France) or nutrient yeast broth (NYB; 20 g/L nutrient broth; 5 g/L yeast extract; Biokar), for 24 h at 28 °C and 200 rpm, when required.

### 2.2. Primer Design

Oligonucleotides targeting conserved regions within the *gyrB* gene were designed manually, based on the alignment of the whole *gyrB* gene sequence (2421 bp in *P. aeruginosa* PA01) of *Pseudomonas* species representing all taxonomic clusters based on the classification made by Gomila et al. [7]. Available sequences were retrieved from the GenBank database (as of September 2017; Table S1), for the 168 *Pseudomonas* species described at LPSN (list of Prokaryotic names with standing in Nomenclature) [41]. The alignment of the final set of 148 *gyrB* full sequences was performed with MEGA7 software [42], using the MUSCLE program of the alignment explorer option [43]. Different candidate primer pairs defining amplicons larger than 1200 bp were tested in silico using AmplifX v.1.7.0 [44] and FastPCR 3.3.21 [45]; the best primer pairs were evaluated in vitro by PCR, using a collection of target strains (Table 1). The properties of the novel chosen primers are shown in Table 2.

### 2.3. PCR Reaction

Polymerase chain reaction conditions for the new primer pair reported in this study were optimized to obtain a single amplicon. Typically, 25 μL-reaction mixtures contained 1× buffer (TransGen Biotech, Beijing, China, containing 2.0 mM MgCl_2_), 0.2 mM deoxyribonucleotide triphosphates (dNTPs) (PB-L, Bernal, Argentina), 0.2 μM of each primer (Invitrogen, Carlsbad, CA, USA), 5% (*v*/*v*) dimethyl sulfoxide (DMSO, Sigma Aldrich, St Louis, Missouri, USA), and 1 U of EasyTaq DNA polymerase (TransGen Biotech, China). The PCR cycle consisted of an initial denaturation step of 5 min at 94 °C, followed by 35 cycles of 0.5 min at 94 °C, 0.5 min at 57 °C, and 1 min at 72 °C; and a final extension step at 72 °C for 5 min. As DNA template, we used 1 μL of a thermal cell lysate of each strain. Thermal lysates were obtained by treating a 2–3 mm colony resuspended in 100 μL of ultrapure H_2_O (PCR grade) at 100 °C for 15 min. After amplification, 10 μL of each reaction was run in 1% agarose gels in 0.5× Tris–borate–EDTA (TBE) at 10 V/cm for 50 min. The gel was stained with ethidium bromide and visualized under UV light.

Polymerase chain reactions with the previously described primers [2,13] were performed with 1 μL of template DNA from thermal lysates of reference pseudomonads from our strain collection (Table 1). We tested two primer combinations, based on the recommended reactions described by Mulet and colleagues [14]. We followed the instructions reported for the reaction mixes and cycling conditions corresponding to each primer pair [2,13] in a final volume of 25 μL. Electrophoresis and staining conditions were the same as described previously.

When corresponding, PCR products from representative pseudomonads were sequenced in Macrogen Inc. (Seoul, Korea) from both ends with each one of the primers designed in this study. Reconstruction of the *gyrB* fragment and control of the sequences’ quality were performed manually with the BlastN tool [48]. Sequences from isolates of our strain collection were submitted to Genbank database, and the corresponding accession numbers are detailed in File S1.

### 2.4. PCR-RFLP

Restriction analysis of the *gyrB* sequences was set up in silico with Serial Cloner v2.6.1 [49] using the amplicon sequences obtained by a virtual PCR reaction run with the designed primers (Table 2) on a group of representative strains as templates (Table 1). A set of classical enzymes was tested to select those ones producing differential RFLP patterns for each reference *Pseudomonas* species. To confirm the in silico results, in vitro PCR-RFLP assays were carried out in a final volume of 20 μL, containing 10 μL of PCR mix and 2 U of the endonuclease *TaqI* (Thermo Fisher, Waltham, MA, USA). Reactions were incubated at 62 °C for 3 h. The restriction products were separated by electrophoresis in 2% agarose gels in 0.5× TBE at 6 V/cm for 2 h. Gels were stained with ethidium bromide and DNA banding patterns were visualized under UV light.

## 3. Results

### 3.1. Primer Pair Design and In Silico Tests

**Figure 1:**
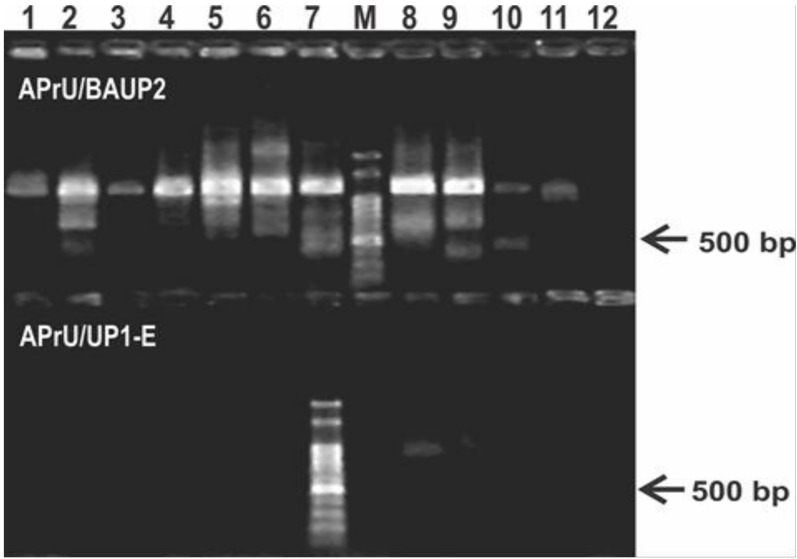
Typical results of the PCR protocol targeting the *gyrB* gene using primers reported in literature. The upper panel of the agarose gel corresponds to PCR reactions using the primer pair APrU/BAUP2 [13]; the lower panel corresponds to PCR reactions using the primer pair APrU/UP1-E [2]. Strains were as follows: 1, *P. stutzeri* ATCC 17588; 2, *P. putida* KT2440; 3, *P. protegens* CHA0; 4, *P. fluorescens* SBW25; 5, *P. syringae* pv. *tomato* DC3000; 6, *P. aeruginosa* PA01; 7; *P. alkylphenolica* KL28; 8, *P. sihuiensis* 2013; 9, *P. donghuensis* SVBP6; 10, *P. chlororaphis* SMMP3; 11, *P.* sp. 1008; 12, negative control (without DNA). Molecular weight markers (M) correspond to the 100-bp DNA ladder (Embiotec, Buenos Aires, Argentina).

Using the most employed *gyrB* oligonucleotides reported in literature, we failed to obtain single bands of the expected size and with uniform yield (Figure 1). We observed a low specificity in the reaction with the APrU/BAUP2 primer pair, and a low yield, or no reaction, with the APrU/UP1-E primer pair (Figure 1). This outcome hampers direct sequencing of the amplicons and forces introduction of a gel extraction step for purification of the expected amplicon before sequencing. These results prompted us to design a novel primer pair targeting *gyrB* based on the high number of *Pseudomonas* genomes that are currently available in public databases (Table S1), and we set up a PCR protocol aiming to obtain a single amplicon suitable for downstream applications, such as RFLP or direct sequencing (Table 2).

**Figure 2:**
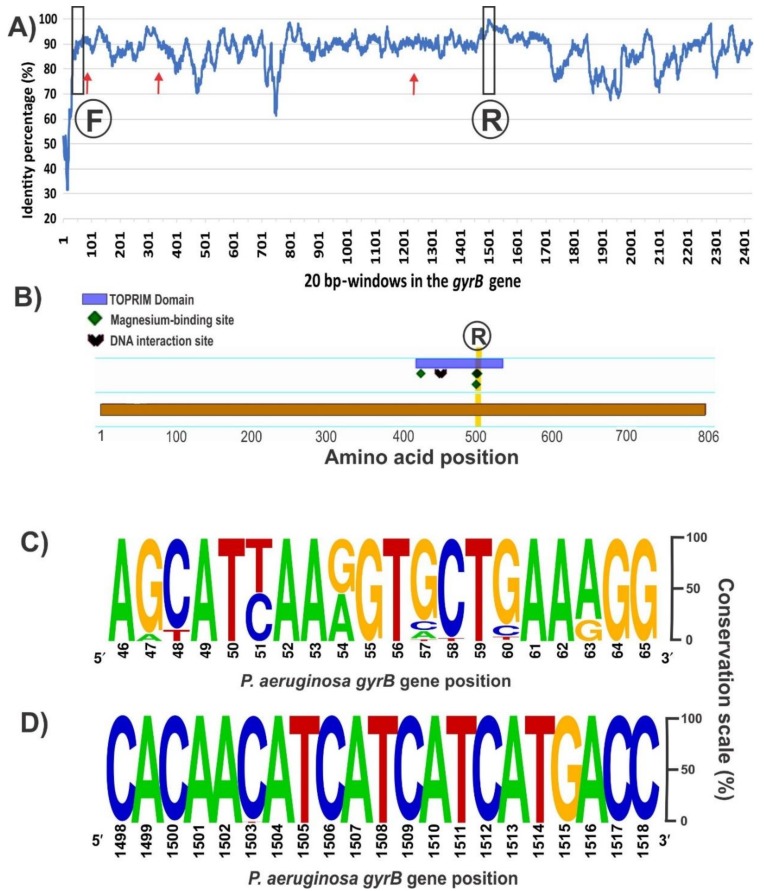
Conservation of the *gyrB* gene among the 148 sequences aligned from all the *Pseudomonas* genomes accessible in Genbank on September 2017 (**A**), domain and conserved sites in the GyrB monomer (**B**), and frequency plots of conserved regions selected for the primers design (**C**,**D**). (**A**) Conservation of the *gyrB* sequence among the 148 genomes analyzed. The percentage of every position was calculated with Jalview software v. 2.1.3 [52] based on the alignment performed in MEGA7 with MUSCLE. Then, an average percentage value was obtained from 20 bp windows, as that is the standard primer size. The positions where the forward (F) and reverse (R) primers were selected are marked. Red arrows show the annealing positions of the previously described forward primers BAUP2 (position 63) and UP-1E (position 335), and the reverse APrU (position 1234); (**B**) GyrB features in the amino acid sequence from *P. aeruginosa* PA01 obtained with the Feature Viewer of UniProtKB database (Q9I7C2). The TOPRIM (topoisomerase-primase) domain contains three key amino acids involved in the binding of two magnesium cations and a DNA interaction site. Positions 500 and 502, where the second magnesium binds, matches the highly conserved nucleotide region selected for the design of the novel reverse primer; (**C**) Forward primer was designed based on this conserved region of 20 bp at the beginning of the *gyrB* gene (between positions 46 and 65 of the sequence from *P. aeruginosa* PA01 strain). As we show in Table 2, positions 51, 54 and 63, where the frequency was distributed between two nucleotides, were replaced by the corresponding partially degenerate IUPAC (International Union of Pure and Applied Chemistry) base codes, trying to avoid fully degenerate bases; (**D**) The reverse primer of 21 bp was chosen as part of a sequence region with the highest identity along the *gyrB* gene. The analyses of (c) and (d) were performed with the Weblogo software v2.8.2 [53].

The sequence alignment of the entire *gyrB* gene (ranging 2412–2424 bp long depending on the species) from the 148 available *Pseudomonas* genomes revealed several conserved regions with over 90% of nucleotide identity distributed along the coding sequence (Figure 2A). A desired aspect for comparative taxonomic purposes is to obtain as much sequence information as possible of the housekeeping gene; to this end, we focused in conserved sequence regions separated by at least 1200 bp. Inspection of the 5′ region of the alignment did not reveal a fully conserved stretch with sufficient length for a forward PCR primer (Figure 2A). From the best candidate regions, the chosen one is shown in Figure 2C, which corresponds to positions 46–65 of the *P. aeruginosa* PA01 *gyrB* allele. Based on this 20 bp stretch, a forward primer containing 3 partially degenerate positions was designed, avoiding the incorporation of fully degenerate bases (Table 2). Towards the 3′ region of the *gyrB* alignment, we found a segment of 21 bp (positions 1498–1518 of the *P. aeruginosa* PA01 *gyrB* allele) that is highly conserved among all retrieved sequences and that is ideal for the reverse primer (Figure 2B,D). In silico analyses of the PCR reaction with AmplifX and FastPCR software confirmed the compatibility of the chosen primer pair and provided the optimal annealing temperatures (Table 2). The expected size for the *gyrB* amplicon would be 1461–1467 bp, depending on the target *Pseudomonas* species (Table 2). Previously, reported oligonucleotides for amplification of the *gyrB* gene in several pseudomonads [2,14], anneal in different regions from those targeted by our newly designed ones, and were expected to generate an amplicon of shorter lengths and less informative content (Figure 2A). Although the forward primer zone has a conservation level similar to the forward primer we designed, the reverse oligonucleotide is in a poorly conserved region of the *gyrB* gene compared with our new reverse primer (Figure 2A).

### 3.2. In Vitro Assay with Target Microorganisms

**Figure 3:**
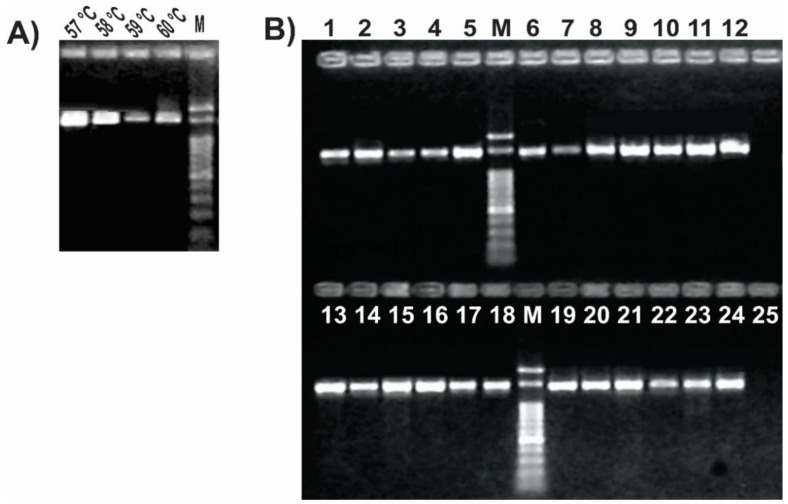
Set up of the PCR protocol targeting *Pseudomonas gyrB* gene. 12.5 μL of each reaction was run in 1% agarose gels in 0.5× Tris–borate–EDTA (TBE) at 10 V/cm for 50 min. (A) Gradient PCR of using a *P. fluorescens* SBW25 lysate as template. Reactions were carried out with primers listed in Table 2 with the annealing temperatures shown above each lane; (B) PCR reactions for the pseudomonads listed in Table 1. Strains were as follows: 1, *P. stutzeri* ATCC 17588; 2, *P. stutzeri* 2014; 3, *P. stutzeri* 2018; 4, *P. putida* KT2440; 5, *P. putida* GR12-2; 6, *P. protegens* CHA0; 7, *P. protegens* Pf-5; 8, *P. fluorescens* 2–79; 9, *P. fluorescens* SBW25; 10, *P. simiae* WCS417; 11, *P. syringae* pv. *tomato* DC3000; 12, *P. syringae* pv. *maculicola* ES4326; 13, *P. aeruginosa* PA01; 14; *P. aeruginosa* Hex1T; 15, *P. aeruginosa* DN; 16, *P. alkylphenolica* KL28; 17, *P. sihuiensis* 2013; 18, *Pseudomonas* sp. 2019; 19, *P. donghuensis* SVBP6; 20, *P. chlororaphis* SMMP3; 21, *P. sihuiensis* N23; 22, *Pseudomonas* sp. CF5; 23, *Pseudomonas* sp. LDe; 24, *P.* sp. 1008; 25, negative control (without DNA). Molecular weight markers (M) correspond to the 100-bp DNA ladder (Embiotec).

We set up the PCR protocol by optimizing the reaction mix and cycling conditions, so that all tested samples resulted in a single PCR product of the expected size (Figure 3). The addition of DMSO to the PCR mix improved the amplification yield and specificity, as previously reported for other protocols [20,50]. A gradient PCR test with annealing temperatures between 49 °C and 57 °C was performed to find the optimal condition where we could obtain a single band and a good yield (Figure S1). After confirming that the amplification was successful at up to 57 °C, an additional PCR gradient between 57 °C and 60 °C was performed to check if we could improve the PCR specificity without losing yield. We found that 57 °C was the optimal annealing temperature (Figure 3A), in agreement with the in silico prediction. Once set up, we confirmed the ability of the optimized PCR protocol using this unique primer pair (Table 2) to amplify a 1.4 kb internal portion of the *gyrB* gene from a range of *Pseudomonas* strains representing most of the different species subgroups (Table 1 and Figure S2). As shown in Figure 3B, all thermal lysates of the tested pseudomonads resulted in a single amplicon of the expected size, without any secondary product. Also, we observed that this reaction protocol allows performing colony PCR instead of preparing thermal lysates or DNA extraction as template (Figure S1). Amplicon sequencing using either of both novel primers allowed us to reconstruct a sequence fragment of around 1400 bp long, thus covering over 95% of the amplicon size obtained in the PCR reaction (File S1). Nonetheless, the *gyrB* amplicon sequences of the environmental isolate *Pseudomonas* sp. N23 [37] and of the clinical isolate *Pseudomonas* sp. 2013, the latter previously assigned to the species *P. mendocina* [20], enabled us to reassign those isolates to the recently described species *P. sihuiensis* [51], as the sequence identity raised 99% with 100% of coverage in the BlastN analysis with the sequence from *P. sihuiensis* KCTC 32246 (Genbank accession number LT629797.1) (Figure S2 and File S1). Thus, in a single PCR round, the newly designed primers provided amplicons that are suitable for direct sequencing and taxonomic analysis.

### 3.3. PCR-RFLP

**Figure 4:**
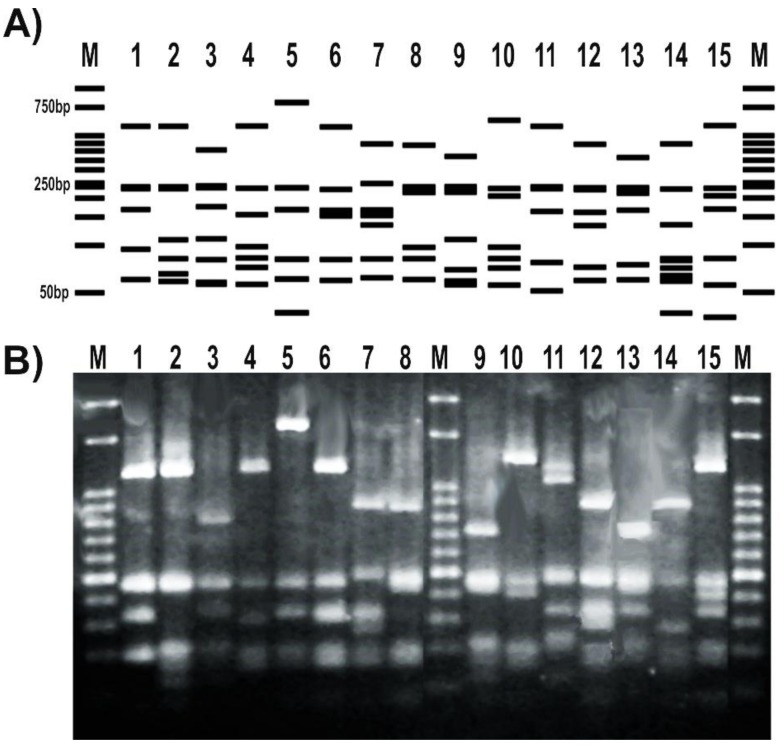
Set up of a PCR-RFLP (restriction fragment length polymorphism) protocol targeting *Pseudomonas gyrB* gene. (**A**) RFLP patterns obtained from the in silico analysis of the amplicons sequenced at Macrogen S.A. and digested with *TaqI*; (**B**) RFLP patterns of representatives from our pseudomonads collection. Strains were as follows: 1, *P. fluorescens* 2–79; 2, *P.* sp. 1008; 3, *P. fluorescens* SBW25; 4, *P. protegens* CHA0; 5, *P. simiae* WCS417; 6, *P. chlororaphis* SMMP3; 7, *P. syringae pv. syringae* DC3000; 8, *P. putida* KT2440; 9, *P. aeruginosa* PA01; 10, *P. alkylphenolica* KL28; 11, *P. stutzeri* ATCC 17588; 12, *P. stutzeri* 2018; 13, *P.* sp. CF5; 14, *P. sihuiensis* N23; 15, *P. donghuensis* SVBP6. Markers (M) are 50-bp DNA ladder (Embiotec).

The RFLP patterns obtained for a subset of tested strains matched the expected profiles deduced from the in silico analysis of *gyrB* sequenced amplicons (Figure 4A). In addition, the RFLP banding patterns obtained after the digestion with *TaqI* were able to distinguish different pseudomonas species (Figure 4B). Moreover, the RFLP profile of different representatives of the same *P. fluorescens* species showed differential patterns with this enzyme (Figure 4).

## 4. Discussion

The *gyrB* sequence is a useful tool for MLSA in bacteria [4,8,9,10,11] that improves the results obtained with classical approaches, such as DNA–DNA hybridization (technically challenging) or 16S ribosomal DNA (rDNA) gene sequencing (nowadays with a low resolving power for some taxa) [10]. Particularly, it is broadly employed for identification and phylogenetic analyses of members of a complex bacterial genus such as *Pseudomonas* [7,54,55]. The taxonomy of pseudomonads is continuously being revised, due to the discovery of new species, the reclassification of some of them, or reorganization of groups [7,56,57,58,59,60]. When we looked for a primer pair available in literature to obtain a single *gyrB* amplicon from diverse *Pseudomonas* isolates, we noticed that there was not a single one that could be employed for the amplification of representatives of the entire genus, but only specific primers for some species or groups [16,17,18,19]. Besides, when we tested published primer pairs, we failed to obtain a single band suitable for direct sequencing (Figure 1). When those oligonucleotides were designed, they were based on amino acid sequences from different bacterial groups [2,12,13,61], and the availability of *gyrB* nucleotide sequences was low. Thus, we considered that a new protocol was needed to improve obtaining a unique and specific band from the *gyrB* gene. A simple and rapid MLSA method could be performed by amplifying and sequencing directly the selected amplicons [10]. Also, a one-step procedure avoids additional investment of laboratory material and person-hours needed to obtain the sequences. Certainly, as many reported primer pairs for MLSA were designed when a much lower number of sequences were available in databases than today, we consider that there might be other housekeeping genes, such as *rpoB*, *rpoD*, *gyrA* or *recA*, whose sequences may require a similar analysis to eventually upgrade their PCR primers for MLSA, to confirm if the recently described *Pseudomonas* species are included as targets.

In silico analyses allowed us to find conserved regions of the *gyrB* gene from available *Pseudomonas* genomes (Figure 2). The region of the gene on which our reverse primer was designed determines a central role in the enzymatic activity of GyrB, thus explaining the strong sequence conservation (Figure 2B). Amino acid residues in positions 500 and 502, whose reverse complement codons are part of our reverse primer (Figure 2D), bind each one a magnesium cation that acts as cofactor, forming salt bridges with both the protein and the DNA [62]. Based on the characteristics of this sequence region, we selected and designed the forward primer to be compatible with. This methodology allowed us to obtain a robust primer pair that specifically amplify a long *gyrB* fragment, avoiding additional steps such as gel extraction and purification (Figure 3). Although members of the *P. pertucinogena* and *P. straminea* groups were not available in our strain collection (Table 1) to carry out the in vitro tests, both computational tools Amplifx and FastPCR confirmed that this newly designed primer pair amplifies the *gyrB* sequences from species included in these groups (Figure S2). Moreover, as we targeted strongly conserved regions to design the oligonucleotides, this primer pair could also be used for amplification of *gyrB* sequences from other Proteobacteria; amplicons with the expected size were obtained in our lab for *Escherichia coli* K12, *Vibrio harveyii* BB170, *Pantoea* spp., *Chromobacterium violaceum* VIR07 and *Salmonella* spp. (Figure S3). Polymerase chain reaction coupled with RFLP is a sensitive and economical approach that could be carried out in any molecular biology laboratory. Several PCR-RFLP assays based on other genes have been previously described as a useful and rapid tool to distinguish environmental and clinical isolates of this bacterial genus, and also to analyze pseudomonads communities under different treatments [20,63,64,65,66,67,68]. Here, we demonstrated that the housekeeping gene *gyrB* is also a suitable target for such kind of comparative taxonomic analyses (Figure 4). This may enhance the applicability of this method as a broad analysis tool, not only for the *Pseudomonas* group [69].

## 5. Conclusions

The *gyrB* gene sequence has become a useful tool for phylogenetic analyses of the *Pseudomonas* genus as part of MLSA approaches [7,14,60]. Polymerase chain reaction primers available in the literature before this study were designed based on the GyrB amino acid sequences of different bacterial taxa, thus they often result in unspecific amplification or lack of amplicons for some pseudomonas isolates (Figure 1). Here, we present a robust novel pair of primers targeting a larger and more informative region of the *gyrB* gene with broad amplification success across the pseudomonads clade (Table 2, Figure 2 and Figure S2). We demonstrated in silico that this primer pair is able to amplify the *gyrB* gene from all the *Pseudomonas* species described at the moment [41]. In vitro, we confirmed the efficiency and specificity of the novel primer pair and of the PCR protocol for a subset of type strains and isolates from the *Pseudomonas* genus (Table 1, Figure 3 and Figures S2). In addition, the *gyrB* amplicons were successfully sequenced from both ends using each of the same primers (Figures S2 and File S1) and served as templates for RFLP assays to distinguish among *Pseudomonas* species (Figure 4).

## Figures and Tables

**Table 1:** *Pseudomonas *strains used to set up the *gyrB* polymerase chain reaction (PCR) protocol in vitro.

Strain Designation	Origin	Reference or Source
*P. aeruginosa *DN	Clinical isolate; Hospital de Niños de La Plata, Argentina	Diego Noseda (CINDEFI—La Plata)
*P. aeruginosa* Hex1T	Hydrocarbon-contaminated soil	[26]
*P. aeruginosa* PAO1	Clinical isolate; human wound; Melbourne, Australia	[27]
*P. alkylphenolica *KL28	Soil from an industrial complex in Changwon, Korea	[28]
*P. chlororaphis *SMMP3	Bulk soil from an agricultural plot under continuous soybean monocropping; Córdoba, Argentina	[29]
*P. donghuensis *SVBP6	Bulk soil from a plot under good agricultural practices, Entre Ríos, Argentina	[29]
*Pseudomonas *sp. 1008	Environmental isolate with high phosphate solubilization ability and used a biofertilizer	Rizobacter Argentina S.A.
*P. fluorescens* 2–79	Soil from an agricultural plot under wheat monocropping; Washington, USA	[30]
*P. fluorescens* SBW25	Phyllosphere from sugar cane; University Farm, Wytham, Oxford, UK	[31]
*P. protegens *CHA0	Tobacco rhizosphere; Payerne, Switzerland	[32]
*P. protegens* Pf-5	Cotton rhizosphere; Texas, USA	[33]
*P. putida* ATCC 17399	Psycrophilic; Western Utilization Research and Development Division of the US. Department of Agriculture; Albany, California	[34]
*P. putida *GR12-2	Psycrophilic bacteria isolated from an unidentified grass in the Canadian High Arctic	[35]
*P. putida* KT2440	Grass rhizosphere, mt-2 derivative; Japan	[36]
*P. sihuiensis* 2013	Clinical isolate, intrahospitalary infection; Buenos Aires, Argentina	Dr. D. Centrón (Faculty of Medicine; UBA, Argentina)
*P. sihuiensis* N23	Laguna Negra, Catamarca, Argentina	[37]
*P. simiae *WCS417	Wheat rhizosphere; Flevoland, The Netherlands	[38]
*P. stutzeri* 2014	Clinical isolate, intrahospitalary infection; Buenos Aires, Argentina	Dr. D. Centrón (Faculty of Medicine; UBA, Argentina)
*P. stutzeri* 2018	Clinical isolate, intrahospitalary infection; Buenos Aires, Argentina	Dr. D. Centrón (Faculty of Medicine; UBA, Argentina)
*P. stutzeri* ATCC 17588	Clinical isolate; human spinal fluid; Copenhagen, Denmark	[34]
*P. syringae *pv. *maculicola* ES4326	Radish rhizosphere; USA	[39]
*P. syringae *pv. *tomato *DC3000	Spontaneous rifampicin resistant strain from the wild-type isolate DC52	[40]
*Pseudomonas* sp. 2019	Clinical isolate, intrahospitalary infection; Buenos Aires, Argentina	Dr. D. Centrón (Faculty of Medicine; UBA, Argentina)
*Pseudomonas* sp. CF5	Feces from extreme high-altitude wetland	Dr. M.E. Farías (LIMLA; Tucumán, Argentina)
*Pseudomonas* sp. LDe	Salina Grande, Jujuy, Argentina	[37]

CINDEFI: Center of Research and Development in Industrial Fermentations; UBA: University of Buenos Aires; LIMLA: Laboratory of Microbiological Research of Andean Lagoons.

**Table 2:** Novel oligonucleotides targeting the *Pseudomonas* spp. *gyrB* gene.

Gene	Amplicon Size (bp) ^a^	Primer	Sequence (5′ → 3′)	Melting Temp. (°C) ^b^	Annealing Temp. (°C)	
					Recommended ^c^	Optimal
*gyrB*	1461–1467	gyrB-F	AGCATYAARGTGCTGAARGG	55.3	55–59	57
		gyrB-R	GGTCATGATGATGATGTTGTG	53.1		

^a^ The size of the gene amplicons was estimated from the sequence of available *Pseudomonas* spp. genomes in GenBank on September 2017. ^b^ Melting temperatures were calculated using the OligoTest tool of the FastPCR software [46], with the Allawi’s thermodynamics parameters, employing the concentration of salts, Mg^+2^, deoxyribonucleotide triphosphates (dNTPs) and dimethyl sulfoxide (DMSO) that are described in Material and Methods. ^c^ Values suggested by the PrimerList tool of the FastPCR software [47].

## Supplementary Materials

The following are available online at http://www.mdpi.com/2409-9279/1/3/24/s1, File S1: *gyrB* sequences obtained after sequencing the PCR products of the reference pseudomonads (Table 1) representing the main taxonomic groups of this genus, Figure S1. Gradient PCR of *gyrB* gene, Figure S2. Evolutionary relationships of *Pseudomonas* species based on the amplicon sequences of the *gyrB* gene, Supplementary Figure S3. Polymerase chain reaction targeting the *gyrB* gene of non-pseudomonads strains of our laboratory collection, with the newly designed primers described in this study, Table S1. Sequences of *gyrB* gene from the *Pseudomonas* species included in the analysis, Table S2. Coverage and accuracy of the sequencing of *gyrB* gene from the *Pseudomonas* strains tested in vitro.
