# Supplementary material for: A Novel Oligonucleotide Pair for Genotyping Members of the Pseudomonas Genus by Single-Round PCR Amplification of the gyrB Gene
Source: Methods Protoc. 2018 Jul 2;1(3):24. doi: 10.3390/mps1030024 (PMC6481054; doi:10.3390/mps1030024)
Supplement: Supplementary file 1 [file mps-01-00024-s001.pdf]

Supplementary Material S1. *gyrB* sequences obtained after sequencing the PCR products of the reference pseudomonads (Table 1) representing the main taxonomic groups of this genus. BlastN analyses of these sequences showed a perfect match between the amplicon-obtained sequences from the reference isolates and those already present in the Genbank database (accession number and locus ID of the matched reference sequence are indicated within parenthesis after the corresponding sequence's name). Sequences from our isolates' collection were submitted to the Genbank database. Accession numbers are indicated at the final of the document.

```
>gyrB_P.florescens_2-79 (JXCQ01000013.1; PFLU3_20420)
TTCGATGTTGTACTCGTCACGGCCATACCGCAGCCCAGCGCTGTGATCAAGGTGCCGACTTCCTGGGAGG
AAATCATCTTGTCAAAACGAGCCTTCTCGACGTTGAGGATCTTACCCTTCAACGGCAGGATAGCCTGGGT
GCGACGGTTGCGACCCTGCTTGGCGGAACCGCCAGCAGAGTCACCTTCCACCAGGTACAGTTCGGAGAGG
GCAGGGTCCTTCTCCTGGCAGTCAGCCAGCTTGCCCGGCAAGCCGGCGATATCCAACGCGCCTTTACGAC
GGGTCATCTCACGGGCCTTACGTGCGGCTTCACGGGCGCGGGCAGCATCGATCATCTTGCCAACGACCAG
CTTGGCTTCGTTCGGGTTTTCCAACAGGAAGTCGGAGAAGTACTTGCCCATTTCCTGTTCAACAGCGGTC
TTCACTTCGGAAGACACCAGCTTGTCTTTGGTCTGGGAGCTGAACTTCGGATCCGGCACCTTGACCGAGA
TGATCGCGGTCAAACCTTCACGGGCATCGTCACCGGTGGTGGCGACTTTATGCTTCTTCGCCAGGCCTTC
CGCTTCGATGTAGGTGTTCAGGTTACGCGTCAGCGCAGAACGGAAACCCACCAAGTGAGTACCGCCATCG
CGCTGCGGAATATTGTTGGTGAAGCACAACAGGTTCTCGTTGAAGCTGTCGTTCCACTGCAGGGCGATTT
CCACGCCGATGCCGTCTTCACGCTGGATGTTGAAGTGGAACACCTGGTTGACTGCAGTCTTGTTGGTGTT
CAGGTATTCAACGAATGCACGCAAGCCGCCTTCGTACTTGAACAGCTCTTCCTTGCCGCTGCGTTCATCC
TTGAGGACGATACCAACACCGGAGTTGAGGAAGGACAGTTCACGAATCCGCTTGGCCAGGATGTCCCAGC
TGAAGTGGATGTTCTTGAAGGTTTCAGCCGATGGCTTGAAGTGGATCTGGGTGCCCGTGGTTTCACTGTC
GCCAACGATTTTCATCGGCTCCTGTGGCACACCGTGGACGTAGGTCTGTTCCCAGATCTTGCCGCTGCGG
CGAACGGTGAGAATCAACTCTTCGGACAGCGCGTTCACTACCGACACACCCACACCGTGCAACCCACCGG
ATACTTTATAGGAGTTGTCGTCGAACTTACCGCCGGCGTGGAGCACGGTCATGATGACCTCTGCCGCCGA
AACGCCTTCTTCCTTGTGCACATCTACCGGGATACCACGGCCGTTGTCGCGCACGGTAATGGACTCATCC
GGGTGGATGATAATGCTGATGTCGTCGCAGTGACCGGCCAGAGCTTCGTCGATGGAGTTATCGACCACCT
CGAACACCATGTGGTGCAGACCGCTACCATCATCGGTGTCGCCAATGTACATACCGGACGT
>gyrB_P.sihuiensis_2013 (LT629797.1; SAMN05216363_3930)
GGATGTACATCGGTGACACTGACGATGGTAGTGGTCTGCACCACATGGTGTTCGAGGTGGTCGACAACTC
GATCGACGAAGCGCTGGCAGGCTACTGCAGTGAAATCACCATCACCATCCACCCGGATGAATCGATCAGT
GTGCGTGATAACGGCCGTGGTATCCCGGTCGACATGCACAAGGAAGAAGGCGTCTCGGCGGCCGAGGTCA
TCATGACCGTGCTTCACGCCGGCGGTAAGTTCGATGACAACAGCTACAAGGTATCCGGCGGCCTGCATGG
CGTGGGTGTCTCGGTGGTGAACGCCCTCTCCAAGGAGCTGGTGCTGACCATCCGCCGTAGCGGCAAAATC
TGGGAACAGACCTACGTGCATGGTGTACCGCAAGCGCCTCTGGCCGCTGTCGGTGACACCGACGGTACCG
GTACGCAGATCCACTTCAAACCGTCCGAAGAGACCTTCGCCAACATCCATTTCAGCTGGGACATCCTGGC
CAAGCGTCTGCGCGAACTGTCCTTCCTCAACTCCGGCGTGGGCATCCTGCTGCGCGACGAGCGCACCGGC
AAGGAAGAGTTGTTCAAGTACGAAGGTGGTCTGAAGGCCTTCGTCGAGTACCTGAACACCAACAAGACCG
TGGTCAACCAGGTGTTCCACTTCAACGTTCAGCGCGAAGAAGACGGCGTTGGTGTCGAAGTTGCCCTGCA
GTGGAACGACAGCTTCAACGAGAACCTGCTTTGCTTCACCAACAACATTCCGCAGCGTGACGGTGGCACG
CACCTGGCCGGTTTCCGTTCCGCCCTCACCCGCCACCTGAACAACTACATCGAGGCCGAAGGCCTGGCCA
AGAAGTTCAAGGTTTCCACCACCGGTGACGATGCCCGCGAAGGTCTGACTGCGATCATCTCGGTCAAGGT
GCCGGACCCGAAATTCAGCTCGCAGACCAAGGACAAACTGGTATCGAGCGAGGTAAAAACTGCGGTCGAA
CAGGAGATGGGCAAGTACTTCTCCGACTTCCTGCTGGAAAACCCCAACGAAGCCAAGGCCGTGGTCGGCA
AGATGATCGATGCCGCGCGTGCCCGCGAAGCGGCTCGTAAAGCCCGTGAAATGACTCGCCGTAAAGGTGC
GCTGGACATTGCCGGCCTGCCCGGCAAACTGGCCGACTGCCAGGAAAAAGACCCGGCGCTGTCCGAACTC
TACATCGTGGAGGGTGACTCCGCGGGCGGTTCTGCCAAGCAGGGCCGTAACCGCAAGACCCAGGCGATCC
TGCCGCTCAAGGGTAAGATCCTCAACGTCGAACGCGCGCGCTTCGACCGCATGATTTCCTCGCAGGAAGT
CGGCACGCTGATCACTGCGCTCGGCTGCGGCATGGCCGTGATGAATACAACATC
>gyrB_P.stutzeri_2014 (CP002622.1; PSTAA_0004)
CTGGCATGTATATCGGCGACACCGATGACGGCACCGGCCTGCACCACATGGTCTTCGAAGTCGTCGATAA
CTCGATCGATGAAGCGCTGGCCGGCTACTGCAGCGATATTTCCATCACCATCCATACCGATGAATCCATC
ACCGTGCGCGACAACGGCCGCGGCATTCCGGTGGATATTCACGAAGAAGGCGTATCGGCAGCCGAGGTCA
TCATGACCGTGCTGCACGCAGGCGGTAAGTTCGACGACAACTCCTACAAGGTATCCGGTGGCCTGCACGG
CGTGGGTGTCTCGGTGGTGAATGCATTGTCCGAGGAGCTGCTGCTGACCATCCGCCGCGAAGGCAAGGTG
TGGGAACAGCTCTATCGCCATGGTGTCCCGCAAGCGCCGCTCGCGGCCGTGGGCGAGACCGATACGTCGG
GCACGCAGATCCATTTCAAACCCTCCGCCGAGACCTTCCAGAACATCCATTTCAGCTGGGACATTCTGGC
CAAGCGTCTTCGCGAACTGTCCTTTCTCAACTCCGGGGTGGGTATCGTCCTGCGTGACGAGCGCACGGCC
AAGGAAGAACTGTTCAAGTACGAAGGCGGCCTCAGCGCCTTCGTCGCCTACCTGAACACCAACAAGACCG
CGGTGAATCAGGTGTTCCACTTCAACGTCCAGCGTGACGATGGCGTCGGCGTCGAAGTCGCGCTGCAGTG
GAACGACAGCTTCAACGAGAACATCCTCTGCTTTACCAACAACATTCCCCAGCGTGACGGCGGCACTCAC
```

CTGGCCGGCTTCCGCTCCGCATTGACGCGTAACCTGAACAACTACATCGAGCAGGAAGGCCTGGCGAAGA
AGCACAAGATCGCCACCACCGGTGACGATGCGCGTGAAGGTCTGACCGCGATCATCTCGGTCAAGGTACC
GGATCCGAAATTCAGTTCCCAGACCAAGGACAAGCTGGTTTCCTCTGAGGTGAAGACCGCGGTGGAACAG
GAGATGGGCAAGTATTTCGCCGACTTCCTGCTGGAGCATCCAAACGAGGCGAAGGCGGTGGTCGGCAAGA
TGATCGACGCTGCCCGCGCCCGTGAGGCTGCGCGCAAGGCACGGGAAATGACCCGGCGCAAAGGCGCGCT
GGATATCGCCGGCCTGCCGGGCAAGCTTGCCGACTGTCAGGAGAAGGATCCTGCGCTGTCCGAACTGTAC
ATCGTGGAGGGTGACTCCGCGGGTGGCTCGGCCAAGCAGGGCCGCAATCGCAAGACCCAGGCGATCCTCC
CGCTCAAGGGCAAGATTCTCAACGTCGAGAAGGCACGCTTCGACAAGATGCTCTCGTCCCAGGACGTCGG
CACGCTGATCACCGCGCTGGGTTGCCGCCTCGCCGCCACGACA
>gyrB_P.stutzeri_2018 (CP002622.1; PSTAA_0004)
TTGTATTCTCGCGGCCGATGCCGCAACCCAGCGCGGTGATCAGCGTGCCGACTTCCTGGGACGAGAGCAT
CTTGTCGAAGCGTGCCTTCTCGACGTTGAGGATCTTGCCCTTGAGCGGGAGGATCGCCTGGGTCTTGCGG
TTGCGGCCCTGCTTGGCCGAGCCACCCGCGGAGTCACCCTCCACGATGTACAGTTCGGACAGCGCAGGAT
CCTTCTCCTGACAGTCGGCAAGCTTGCCCGGCAGGCCGGCGATATCCAGCGCACCTTTGCGCCGGGTCAT
TTCCCGTGCCTTGCGCGCAGCCTCACGGGCGCGGGCAGCGTCGATCATCTTGCCGACTACCGCCTTCGCC
TCGTTTGGATGCTCCAGCAGGAAGTCGGCGAAGTACTTGCCCATCTCCTGTTCCACCGCGGTCTTCACCT
CGGAGGAAACCAGCTTGTCCTTGGTCTGGGAACTGAACTTCGGATCCGGCACCTTGACCGAGATGATCGC
GGTCAGACCTTCACGCGCATCGTCACCGGTGGTGGCGATCTTGTGCTTCTTCGCCAGGCCTTCCTGCTCG
ATGTAGTTGTTCAGGTTACGCGTCAGTGCGGAGCGGAAGCCGGCCAGGTGAGTGCCGCCGTCACGCTGGG
GTATGTTGTTGGTAAAGCAGAGGATGTTCTCGTTGAAGCTGTCGTTCCACTGCAGCGCGACTTCGACGCC
GACGCCATCGTCACGCTGGACGTTGAAGTGGAACACCTGATTCACCGCGGTCTTGTTGGTGTTCAGGTAG
GCGACGAAGGCGCTGAGGCCGCCTTCGTACTTGAACAGTTCTTCCTTGGCCGTGCGCTCGTCGCGCAGAA
CGATACCCACCCCGGAGTTGAGAAAGGACAGTTCGCGAAGACGCTTGGCCAGAATGTCCCAGCTGAAATG
GATGTTCTGGAAGGTCTCGGCGGAGGGTTTGAAATGGATCTGCGTGCCCGACGTATCGGTCTCGCCCACG
GCCGCGAGCGGCGCTTGCGGGACACCATGGCGATAGAGCTGTTCCCACACCTTGCCTTCGCGGCGGATGG
TCAGCAGCAGCTCCTCGGACAATGCATTCACCACCGAGACACCCACGCCGTGCAGGCCACCGGATACCTT
GTAGGAGTTGTCGTCGAACTTACCGCCTGCGTGCAGCACGGTCATGATGACCTCGGCTGCCGATACGCCT
TCTTCGTGAATATCCACCGGAATGCCGCGGCCGTTGTCGCGCACGGTGATGGATTCATCGGTATGGATGG
TGATGGAAATATCGCTGCAGTAGCCGGCCAGCGCTTCGTCAATCGAGTTATCGACGACTTCGAAGACCAT
GTGGTGCAGGCCGGTGCCGTCATCGGTGTCGCCGATATACATGCCAGGC
>gyrB_Pseudomonas_sp._CF5 (CP013987.1; APT59_00155)
CCGGCATGTACATCGGCGACACCGATGACGGCACCGGCCTGCACCACATGGTCTTCGAGGTGGTGGACAA
CTCCATCGACGAGGCCCTGGCCGGCTTCTGCAGCGAGATCAGCATCACCATCCACATGGACGAATCCATC
ACCGTGCGCGACAACGGTCGCGGCATCCCGGTGGATATCCACAAGGAGGAAGGCGTTTCCGCAGCCGAGG
TCATCATGACCGTGCTGCACGCCGGCGGTAAGTTCGATGACAACACCTACAAGGTGTCCGGCGGTCTGCA
CGGCGTGGGCGTCTCGGTGGTGAACGCCCTGTCCGAAGAGCTGCGACTGACCATTCGCCGCGCCGGCCAG
GTATGGGAGCAGGTCTATGTGCATGGCGTGCCCCAGGCGCCGCTGGCGGTGATCGGCGAGACCGACACCA
CCGGTACCCAGGTGCACTTCAAGCCGTCCGCCGAGACCTTCAGCAACATCCAGTTCAGCTGGGACATCCT
GGCCAAGCGGATTCGCGAGCTGTCCTTCCTCAACTCCGGCGTCGGCATCGTCCTGCGCGACGAGCGCAGC
GGCAAGGAAGAGCTGTTCAAGTACGAAGGCGGCCTGCGTGCCTTCGTCGACTACCTCAACACCAACAAGA
CCGCGGTGAACGAGGTGTTCCATTTCCAGGTGCAGCGCGAAGACGATGGCGTCGGCGTGGAAGTGGCCCT
GCAGTGGAACGACAGCTTCAACGAGAACATCCTCTGCTTCACCAACAACATTCCCCAGCGCGATGGCGGT
ACCCACCTGGCGGGCTTCCGCTCGGCTCTGACGCGCAACCTGAACAACTACATCGAGGCCGAGGGCCTGG
CCAAGAAGTTCAAGGTGGCCACCACCGGTGACGACGCCCGCGAAGGCCTGACCGCCATCATCTCGGTCAA
GGTGCCGGATCCCAAGTTCTCCTCCCAGACCAAGGACAAGCTGGTCTCCTCCGAGGTGAAGACCGCGGTG
GAACAGGAGATGGGCAAGTACTTCTCCGACTTCCTGCTGGAGAATCCCAACGAGGCCAAGGCGGTGGTCG
GCAAGATGATCGACGCGGCCCGGGCCCGGGAAGCGGCGCGCAAGGCGCGGGAGATGACCCGCCGCAAGGG
CGCCCTGGACATCGCCGGTCTGCCCGGCAAGCTGGCGGACTGCCAGGAAAAGGACCCGGCGCTGTCCGAA
CTCTACATCGTGGAGGGTGACTCCGCCGGTGGTTCGGCCAAGCAGGGCCGCAACCGGCGCACCCAGGCGA
TCCTGCCGCTCAAGGGCAAGATCCTCAACGTCGAGAAGGCCCGCTTCGACAAGATGCTCTCCTCCCAGGA
GGTAGGCACCCTGATCACCGCGCTGGGCTGCGGTATCGGTCGCGAAGAGTCAA
>gyrB_P.protegens_CHA0 (CP003190.1; PFLCHA0_c00040)
CCGGTATGTACATTGGCGACACCGATGACGGCAGCGGTCTGCACCACATGGTGTTCGAGGTGGTCGATAA
CTCCATCGACGAAGCGCTGGCCGGTCATTGCGACGACATCAGCATCATCATCCATCCGGATGAATCCATC
ACCGTGCGCGACAACGGTCGCGGCATCCCGGTCGATGTGCATAAAGAAGAAGGCGTCTCCGCGGCAGAGG
TCATCATGACCGTGCTCCACGCCGGCGGTAAGTTCGACGACAACTCCTACAAGGTCTCCGGCGGTCTGCA
CGGCGTAGGTGTATCGGTAGTGAACGCCCTGTCCGAGGAGCTGATCCTCACCGTGCGCCGTAGCGGCAAG
ATCTGGGAACAGACCTATGTCCACGGTGTTCCGCAAGAGCGGATGAAAATCGTTGGTGACAGCGAAACCA
CCGGTACCCAGATCCACTTCAAGCCTTCGGCTGAAACCTTCAAGAACATCCACTTCAGCTGGGACGTCCT
GGCCAAGCGGATCCGTGAACTGTCCTTCCTCAACTCCGGTGTCGGCATCGTCCTCAAGGACGAGCGCAGC
GGCAAGGAAGAGCTGTTCAAGTACGAAGGCGGCCTGCGGGCATTCGTTGAATACCTGAACACCAACAAGA
CTGCGGTCAACCAGGTGTTCCACTTCAACATCCAGCGTGAAGACGGCATCGGCGTGGAAATCGCCTTGCA
GTGGAACGACAGCTTCAACGAGAACCTGTTGTGCTTCACCAACAACATTCCTCAGCGCGACGGCGGTACC
CACCTGGTGGGCTTCCGTTCGGCCCTGACCCGTAACCTGAACAACTACATCGAGCAGGAAGGCCTGGCCA

AGAAACACAAGGTCGCCACCACTGGTGACGACGCTCGTGAAGGCCTGACCGCGATCATCTCGGTGAAGGT
GCCGGATCCGAAGTTCAGCTCCCAGACCAAGGACAAGCTGGTTTCTTCCGAAGTGAAGACCGCGGTCGAA
CAGGAGATGGGCAAGTACTTCTCCGACTTCCTGCTGGAAAACCCCAACGAAGCCAAGTTGGTGGTGGGCA
AGATGATCGACGCTGCCCGCGCTCGTGAAGCGGCGCGTAAAGCCCGTGAAATGACCCGTCGCAAAGGCGC
GCTGGATATCGCCGGCCTGCCGGGCAAACTGGCGGACTGCCAGGAAAAGGATCCGGCCCTGTCCGAACTC
TACCTGGTGGAAGGTGACTCTGCTGGCGGTTCCGCCAAGCAGGGCCGCAACCGCAAGACCCAGGCGATCC
TGCCGCTCAAGGGCAAGATCCTCAACGTCGAGAAGGCACGCTTCGACAAGATGATCTCCTCGCAAGAGGT
GGGCACCCTGATCACCGCGCTGGGCTGCGGCATCGGCCGTGAAGAGTACAACATCGCCCGCAACCCCCGG
>gyrB_P.syringae_pv._syringae_DC3000 (AE016853.1; PSPTO_0004)
CGCAGGCGTCCGGATGTACATCGGTGATACAGACGATGGCAGCGGTCTGCACCATATGGTGTTCGAGGTG
GTTGATAACTCGATCGACGAAGCATTGGCAGGCCATTGCGACGACATCAGCATCATCATCCATCCAGACG
AATCTATCACCGTGCGCGACAACGGCCGCGGTATTCCGGTAGACGTGCATAAAGAAGAAGGCGTGTCGGC
AGCCGAAGTCATCATGACCGTGCTCCACGCGGGTGGTAAGTTCGATGACAACTCCTACAAAGTATCCGGC
GGTTTGCACGGTGTAGGTGTTTCCGTGGTCAACGCCCTTTCAGAACTTCTGTTGCTGACAGTGCGTCGCA
GCGGCAAGATCTGGGAACAGACCTACATTCACGGTGTTCCACAAGAACCGATGAAAATCGTCGGCGAAAG
CGACAGCACCGGCACCCAGATTCACTTCAAGCCATCTGCTGAGACCTTCAAGAATATCCACTTCAGCTGG
GACATTCTGGCCAAGCGGATCCGTGAACTGTCGTTCCTGAACTCCGGTGTTGGCATCGTCCTCAAGGACG
AGCGTAGCGGCAAGGAAGAACTGTTCAAGTACGAAGGCGGTTTGCGTGCGTTCGTTGAATACCTGAACAC
CAACAAGACGCCGGTCAACGAAGTGTTCCACTTCAATGTCCAGCGCGATGACGGTATTGGCGTCGAGATT
GCCCTGCAGTGGAACGACAGCTTCAACGAGAACCTGTTGTGCTTCACCAACAACATTCCACAGCGCGATG
GCGGCACTCACCTGGTGGGGTTCCGATCCGCACTGACGCGTAACCTGAACAACTACATCGAGCAGGAAGG
TCTGGCCAAGAAGCACAAGGTCGCGACCACCGGTGACGATGCGCGTGAAGGTCTGACTGCAATTATCTCG
GTCAAAGTACCTGATCCGAAGTTCAGCTCGCAGACCAAAGACAAGCTGGTTTCGTCCGAAGTCAAAACTG
CAGTCGAACAGGAAATGGGCAAGTACTTCTCCGACTTCCTGCTGGAAAACCCGAACGAAGCCAAAGCCGT
CGTCGGCAAGATGATCGACGCAGCCCGTGCCCGTGAAGCCGCGCGCAAGGCCCGTGAGATGACTCGCCGT
AAAGGCGCGCTGGACATCGCCGGCTTGCCAGGCAAACTGGCTGACTGCCAGGAAAAAGACCCTGCCCTCT
CCGAACTGTACCTGGTGGAAGGGGACTCTGCTGGCGGATCAGCCAAGCAGGGACGTAACCGTAGAACCCA
GGCCATCCTGCCTCTTAAGGGCAAGATTCTGAACGTTGAAAAAGCACGCTTCGACAAGATGATCTCTTCT
CAGGAAGTGGGCACCTTGATCACCGCGCTGGGCTGTGGCATGGCCTGCGAAGAGTACAACATCGACA
>gyrB_P.alkylphenolica_KL28 (CP009048.1; PSAKL28_00120)
CCGGTATGTACATTGGCGACACCGATGATGGTAGCGGCCTGCACCACATGGTGTTCGAGGTGCTCGATAA
CTCGATCGACGAAGCACTCGCCGGCCACTGTGATGACATTACCGTAACTATCCACCCGGACGAATCCATC
AGTGTTCGCGACAACGGTCGCGGTATCCCAGTCGACGTGCATAAAGAGGAAGGCGTATCCGCCGCCGAGG
TCATCATGACCGTCCTGCACGCTGGCGGTAAGTTCGACGACAACTCCTACAAAGTATCCGGCGGTCTGCA
CGGTGTAGGCGTGTCTGTGGTTAACGCCCTCTCCGAAGAGCTGATCCTGACTGTTCGCCGTAGCGGCAAG
ATCTGGGAACAGACCTATGTTCACGGTGTTCCGCAAGCTCCGATGGCCATCGTCGGTGACAGTGAAACCA
CCGGTACCCATATTCACTTCAAGCCGTCGTCCGAAACCTTCAAGAACATCCACTTCAGCTGGGACATCCT
GGCCAAGCGGATTCGTGAACTGTCGTTCCTCAACTCTGGCGTCGGCATCCTCTTGAAGGACGAGCGTTCG
GGCAAGGAAGAGTACTTCAAGTACGAAGGCGGTCTGCGTGCCTTCGTTGAGTACCTGAACACCAACAAGA
CCCCGGTCAACCAGGTGTTCCACTTCACCGTTCAGCGTGAAGACGGTGTGGGCGTGGAAATCGCTCTGCA
ATGGAACGACAGCTTCAATGAGAACCTGTTGTGCTTCACCAACAACATTCCGCAGCGCGACGGTGGTACC
CACCTGGTCGGCTTCCGCTCGGCGTTGACCCGTAACCTGAACAACTACATTGAGCAGGAAGGTCTGGCCA
AGAAGAACAAGGTTTCGACCACCGGCGACGACGCCCGCGAAGGTTTGACCGCGATCATTTCGGTGAAGGT
GCCAGATCCTAAGTTCAGCTCGCAGACCAAGGACAAGCTGGTTTCTTCGGAAGTGAAAACTGCCGTGGAA
CAGGAAATGGGCAAGTACTTCTCCGACTTCCTGCTGGAGAACCCGAACGAAGCCAAGGCGGTTGTCGGCA
AGATGATCGACGCCGCGCGTGCTCGTGAAGCAGCGCGCAAGGCGCGGGAAATGACTCGCCGTAAAGGCGC
GCTGGATATTGCTGGTTTGCCAGGCAAACTGGCGGACTGCCAGGAGAAGGACCCTGCCCTTTCCGAACTG
TACCTGGTGGAGGGTGACTCCGCAGGTGGTTCGGCCAAGCAAGGTCGTAACCGTAAGACCCAGGCCATCC
TGCCGCTCAAGGGTAAGATCCTCAACGTCGAGAAAGCACGTTTCGACAAGATGATCTCGTCCCAGGAAGT
TGGCACCCTGATTACCGCACTGGGCTGTGGTATCGGTCGCGAAGAGTACAACATCGACA
>gyrB_P.putida_KT2440 (AE015451.2; PP_0013)
AGCGTCCCGGCATGTACATTGGCGACACCGATGATGGTAGTGGCCTGCACCACATGGTCTTCGAGGTGGT
CGACAACTCGATCGACGAAGCCCTCGCCGGTCACTGCGATGACATTACCGTCATCATCCACCCGGACGAA
TCTATCAGTGTGCGCGACAACGGTCGCGGCATTCCGGTCGATGTGCATAAGGAAGAAGGCGTTTCCGCAG
CCGAGGTCATCATGACTGTGCTGCACGCCGGCGGTAAGTTTGACGACAACTCCTACAAAGTATCCGGCGG
TCTGCACGGTGTAGGTGTGTCGGTTGTGAACGCCCTGTCCGAGAAGCTGGTTTTGACTGTTCGCCGTAGC
GGCAAGATCTGGGAACAGACTTACGTTCACGGTGTTCCACAAGCGCCCATGGCGGTTGTCGGTGACAGTG
AAACCACGGGTACCCACATCCACTTCAAGCCATCGGCTGAAACCTTCAAGAACATTCACTTCAGCTGGGA
CATCCTGGCCAAGCGCATCCGCGAGCTGTCGTTCCTCAACTCGGGCGTTGGCATTCTGCTGAAGGATGAG
CGCAGCGGTAAGGAAGAGTTCTTCAAGTACGAAGGCGGTCTGCGTGCGTTCGTCGAGTACTTGAACACCA
ACAAGACGCCGGTCAACTCCCAGGTGTTCCACTTCAACGTTCAGCGTGACGATGGCGTGGGTGTTGAAGT
CGCCCTGCAATGGAACGACAGCTTCAACGAAAACCTGCTGTGCTTTACCAACAATATTCCGCAGCGTGAT
GGCGGTACCCACCTGGTGGGTTTCCGTTCCTCGCTGACCCGTAGCCTTAACAGCTACATCGAGCAGGAAG
GCCTGGCCCAAGAAGAACAAGGTGGCAACCACTGGCGACGACGCCCGTGAAGGCCTGACCGCGATCATCT

CGGTGAAGGTACCGGACCCGAAGTTCAGCTCGCAGACCAAGGACAAGCTGGTCTCCTCGGAGGTGAAAAC
CGCCGTGGAACAGGAGATGAACAAGTACTTCGCCGATTTCCTCCTGGAAAACCCGAACGAGGCGAAGGCC
GTCGTTGGCAAGATGATCGACGCCGCTCGCGCCCGTGAAGCCGCCCGTAAAGCCCGTGAGATGACCCGCC
GTAAAGGTGCGCTGGATATCGCGGGTCTGCCGGGCAAGCTGGCCGACTGCCAAGAGAAGGATCCTGCTCT
CTCCGAACTGTACCTGGTGGAGGGTGACTCCGCGGGTGGCTCGGCCAAGCAAGGCCGCAACCGTCGTACC
CAGGCGATCTTGCCGCTGAAGGGTAAAATCCTCAACGTCGAGAAAGCGCGCTTCGACAAGATGATTTCGT
CCCAGGAAGTGGGCACGCTGATCACTGCGCTGGGCTGTGGCATCGGCCGCGAAGAGTACAACATCGACA
>gyrB_P.sihuiensis_N23(LT629797.1; SAMN05216363_3930)
GCAAACGTCCCGGGATGTACATCGGTGACACTGACGATGGTAGTGGTCTGCACCACATGGTGTTCGAGGT
GGTCGACAACTCGATCGACGAAGCGCTGGCAGGCTACTGCAGTGAAATCACCATCACCATCCACCCGGAT
GAATCGATCAGTGTGCGCGATAACGGCCGTGGTATCCCGGTCGACATGCACAAGGAAGAAGGCGTCTCGG
CGGCCGAGGTCATCATGACCGTGCTTCACGCCGGCGGTAAGTTCGATGACAACAGCTACAAGGTATCCGG
CGGCCTGCATGGCGTGGGTGTCTCGGTGGTGAACGCCCTCTCCAAGGAGCTGGTGCTGACCATCCGCCGT
AGCGGCAAGATCTGGGAACAGACCTACGTGCATGGTGTACCGCAAGCGCCTCTGGCCGCTGTCGGTGACA
CCGACGGTACCGGTACGCAGATCCACTTCAAACCGTCCGAAGAGACCTTCGCCAACATTCACTTCAGCTG
GGACATCCTGGCCAAGCGTCTGCGCGAACTGTCCTTCCTCAACTCCGGCGTGGGCATCCTGCTGCGCGAC
GAGCGCACCGGCAAGGAAGAGCTGTTCAAGTACGAAGGTGGTCTGAAGGCTTTCGTTGAGTACCTGAACA
CCAACAAGACCGTGGTCAACCAGGTGTTCCACTTCAACGTTCAGCGCGAAGAAGACGGCGTTGGTGTCGA
AGTTGCCCTGCAGTGGAACGACAGCTTCAACGAGAACCTGCTTTGCTTCACCAACAACATTCCGCAGCGT
GACGGTGGTACGCACCTGGCCGGTTTCCGTTCCGCCCTCACCCGCCACCTGAACAACTACATCGAGGCCG
AAGGCCTGGCCAAGAAGTTCAAGGTTTCCACCACCGGTGACGATGCCCGCGAAGGTCTGACTGCGATCAT
CTCGGTCAAGGTGCCGGACCCGAAATTCAGCTCGCAGACCAAGGACAAACTGGTATCGAGCGAGGTAAAA
ACTGCGGTCGAACAGGAGATGGGCAAGTACTTCTCCGACTTCCTGCTGGAAAACCCCAACGAAGCCAAGG
CCGTGGTCGGCAAGATGATCGATGCCGCGCGTGCCCGCGAAGCGGCTCGTAAAGCCCGTGAAATGACTCG
CCGTAAAGGTGCACTGGACATTGCCGGCCTGCCCGGCAAACTGGCCGACTGCCAGGAAAAAGACCCGGCG
CTGTCCGAACTCTACATCGTGGAGGGTGACTCCGCGGGCGGTTCTGCCAAGCAGGGCCGTAACCGCAAGA
CCCAGGCGATCCTGCCGCTCAAGGGCAAGATCCTCAACGTCGAACGCGCGCGCTTCGACCGCATGATTTC
CTCGCAGGAAGTCGGCACGCTGATCACTGCGCTCGGCTGCGGCATGGCCGTGATGACTACAACATCGATC
AA
>gyrB_P.aeruginosa_PA01 (AE004091.2; PA0004)
CCCGGCATGTACATCGGCGACACCGACGATGGCACCGGTCTGCACCACATGGTGTTCGAGGTGGTGGATA
ACTCCATCGACGAAGCGCTGGCCGGTTACTGCAGCGAAATCAGCATCACCATCCATACGGATGAGTCGAT
CACTGTCCGCGACAATGGACGCGGTATTCCGGTGGATATCCACAAGGAAGAAGGGGTTTCTGCGGCGGAA
GTGATCATGACCGTCCTCCACGCCGGCGGCAAGTTCGACGACAACACCTACAAGGTGTCCGGCGGCTTGC
ACGGTGTGGGCGTCTCGGTGGTGAACGCGCTGTCCCATGAACTACGCCTGACCATCCGTCGCCACAACAA
GGTCTGGGAACAGGTCTACCACCACGGCGTTCCGCAGTTCCCACTGCGCGAAGTGGGCGAGACCGATGGC
TCCGGCACCGAAGTTCACTTCAAGCCGTCCCCGGAGACCTTCAGCAACATCCACTTCAGTTGGGACATCC
TGGCCAAGCGCATCCGCGAGCTGTCCTTCCTCAACTCCGGCGTCGGCATCCTGCTGCGCGACGAGCGTAC
CGGCAAGGAGGAGCTGTTCAAGTACGAAGGCGGTCTGAAGGCCTTCGTCGAGTACCTGAACACCAACAAG
ACCGCGGTGAACGAGGTATTCCACTTCAACGTCCAGCGTGAAGAGGACGGCGTGGGTGTGGAAGTCGCCT
TGCAGTGGAACGACAGCTTCAACGAGAACCTGCTCTGCTTCACCAACAACATCCCGCAGCGTGACGGCGG
CACCCACCTGGCCGGTTTCCGTTCGGCGCTGACGCGTAACCTGAACAACTACATCGAGGCCGAAGGCCTG
GCGAAGAAGTTCAAGATCGCCACCACCGGCGACGATGCCCGCGAAGGCCTCACCGCGATCATCTCGGTGA
AGGTACCGGACCCGAAGTTCAGCTCGCAGACCAAGGACAAGCTGGTCTCCTCCGAGGTGAAGACTGCGGT
GGAACAGGAGATGGGCAAGTACTTCGCCGACTTCCTGCTGGAGAATCCCAACGAAGCCAAGGCCGTGGTC
GGCAAGATGATCGACGCCGCCCGTGCCCGCGAGGCCGCGCGCAAGGCGCGCGAGATGACCCGCCGCAAGG
GCGCGCTGGACATCGCCGGCCTGCCCGGCAAACTGGCCGATTGCCAGGAAAAGGACCCGGCGCTCTCCGA
ACTGTACATCGTGGAGGGTGACTCCGCGGGCGGTTCCGCCAAGCAGGGCCGCAATCGCCGGACCCAGGCG
ATCCTGCCGCTCAAGGGCAAGATCCTCAACGTCGAAAAGGCGCGCTTCGACAAGATGCTCTCCTCCCAGG
AGGTCGGTACGCTGATCACCGCCCTGGGCTGTGGCATCGGCCGCGA
>gyrB_P.stutzeri_ATCC_17588 (CP002881.1; PSTAB_0004)
CATGTATATCGGCGACACCGATGACGGCACCGGCCTGCACCACATGGTCTTCGAAGTCGTCGATAACTCG
ATCGATGAAGCGCTGGCCGGCTACTGCAGCGATATTTCCATCACCATCCATACCGATGAATCCATCACCG
TGCGCGATAACGGCCGCGGCATTCCGGTGGATATTCACGAAGAAGGCGTATCGGCAGCCGAGGTCATCAT
GACCGTGCTGCACGCAGGCGGTAAGTTCGACGACAACTCCTACAAGGTATCCGGTGGCCTGCACGGCGTG
GGTGTCTCGGTAGTGAATGCATTGTCCGAGGAGCTGCTGCTGACCATCCGCCGCGAAGGCAAGGTGTGGG
AACAGCTCTATCGCCATGGTGTCCCGCAAGCGCCGCTCGCGGCCGTGGGCGAGACCGATACGTCGGGCAC
GCAGATCCATTTCAAACCCTCCGCCGAGACCTTCCAGAACATCCATTTCAGCTGGGACATTCTGGCCAAG
CGTCTCCGCGAACTGTCCTTTCTCAATTCCGGCGTGGGTATCGTTCTGCGCGACGAACGCACGGCCAAGG
AAGAACTGTTCAAGTACGAAGGCGGCCTCAGCGCCTTCGTCGCCTACCTGAACACCAACAAGACCGCGGT
GAATCAGGTGTTCCACTTCAACGTCCAGCGTGACGATGGCGTCGGCGTGGAAGTCGCGCTGCAGTGGAAC
GACAGCTTCAACGAGAACATCCTCTGCTTTACCAACAACATTCCCCAGCGTGACGGCGGCACTCACCTGG
CCGGCTTCCGCTCCGCACTGACGCGTAACCTGAACAACTACATCGAGCAGGAAGGCCTGGCGAAGAAGCA
CAAGATCGCCACCACCGGTGACGATGCGCGTGAAGGTCTGACCGCGATCATCTCGGTCAAGGTGCCGGAT

CCGAAGTTCAGTTCCCAGACCAAGGACAAGCTGGTTTCCTCCGAGGTGAAGACCGCGGTGGAACAGGAGA
TGGGCAAGTACTTCGCCGACTTCCTGCTGGAGCATCCAAACGAGGCGAAGGCGGTAGTCGGCAAGATGAT
CGACGCTGCCCGCGCCCGTGAGGCTGCGCGCAAGGCACGGGAAATGACCCGGCGCAAAGGCGCGCTGGAT
ATCGCCGGCCTGCCGGGCAAGCTTGCCGACTGTCAGGAGAAGGATCCTGCGCTGTCCGAACTGTACATCG
TGGAGGGTGACTCCGCGGGTGGCTCGGCCAAGCAGGGCCGCAATCGCAAGACCCAGGCGATCCTCCCGCT
CAAGGGCAAGATTCTCAACGTCGAGAAGGCACGCTTCGACAAGATGCTCTCGTCCCAGGAAGTCGGCACG
CTGATCACCGCGCTGGGTTGCGGCATCGGCCGCGAGGAATACA
>gyrB_Pseudomonas_sp._1008 (CP019856.1; B1R45_00020)
CCGGTATGTACATTGGCGACACTGATGACGGTAGCGGTCTGCACCACATGGTGTTCGAGGTGGTCGACAA
CTCCATCGACGAAGCTCTGGCCGGTCACTGCGACGACATCAGCATTATCATCCACCCGGATGAGTCGATT
ACCGTACGCGACAACGGTCGCGGCATTCCGGTAGACGTGCACAAAGAAGAAGGCGTTTCGGCGGCAGAGG
TCATCATGACCGTGCTCCACGCTGGCGGTAAGTTCGACGACAACTCCTATAAAGTCTCCGGCGGTTTGCA
CGGTGTGGGCGTTTCGGTAGTGAACGCACTGTCCGAAGAGCTGATCCTGACTGTGCGCCGCAGCGGCAAG
ATCTGGGAACAGACCTACGTTCACGGCGTTCCACAAGAACCGATGAAAATCGTCGGCGACAGTGAAACCA
CCGGCACCCAGATTCACTTCAAGCCATCGGCTGAAACCTTCAAGAATATTCACTTCAGCTGGGACATCCT
GGCCAAGCGTATTCGTGAACTGTCGTTCCTTAACTCCGGCGTGGGTATCGTCCTCAAGGACGAGCGCAGC
GGCAAGGAAGAGCTGTTCAAGTACGAAGGCGGCTTGCGTGCGTTCGTTGAATACCTGAACACCAACAAGA
CTGCGGTCAACCAGGTGTTCCACTTCAACATCCAGCGTGAAGACGGCATTGGCGTGGAAATCGCCTTGCA
GTGGAACGACAGCTTCAACGAGAACCTGTTGTGCTTCACCAACAACATTCCTCAGCGCGATGGCGGTACT
CACCTGGTGGGTTTCCGTTCGGCACTGACGCGTAACCTGAACACCTACATCGAAGCCGAAGGCCTGGCGA
AGAAGCATAAAGTTGCCACCACCGGTGACGATGCCCGCGAAGGCTTGACCGCGATTATCTCGGTGAAAGT
ACCGGATCCGAAGTTCAGCTCCCAGACCAAAGACAAGCTGGTGTCTTCCGAAGTGAAGACCGCAGTGGAG
CAGGAGATGGGCAAGTACTTCTCCGACTTCCTGCTGGAGAACCCGAACGAAGCCAAGTTGGTCGTCGGCA
AGATGATCGACGCTGCACGTGCCCGTGAAGCCGCGCGTAAGGCCCGTGAAATGACCCGTCGTAAAGGCGC
GTTGGACATTGCCGGCCTGCCGGGCAAACTCGCTGACTGCCAGGAGAAGGACCCTGCCCTCTCCGAACTG
TACCTGGTGGAAGGTGACTCTGCTGGCGGTTCCGCCAAGCAGGGTCGTAACCGTCGCACCCAGGCCATCC
TGCCGTTGAAGGGTAAGATCCTCAACGTCGAGAAGGCGCGCTTCGACAAGATGATTTCTTCCCAGGAAGT
TGGCACCTTGATCACGGCGTTGGGTTGCGGTATCGGTCGTGACGAATACAACATCGA
>gyrB_P.fluorescens_SBW25 (AM181176.4; PFLU_0004)
CCGGTATGTACATTGGCGACACTGATGACGGTAGCGGTCTGCACCACATGGTGTTCGAGGTGGTCGACAA
CTCCTCGACGAAGCTCTGGCCGGTCACTGCGACGACTCAGCATTATCATCCACCCGGATGAGTCGATTAC
TGTGCGCGACAACGGTCGCGGCATTCCGGTAGACGTACACAAAGAAGAAGGCGTTTCGGCGGCAGAGGTC
ATCATGACCGTGCTTCACGCCGGCGGTAAGTTCGACGACAACTCCTATAAAGTCTCCGGTGGTTTGCACG
GCGTGGGCGTGTCGGTTGTGAACGCGCTGTCCGAAGAACTGATTCTGACGGTTCGCCGTAGCGGCAAGAT
CTGGGAACAGACTTACGTCCACGGCGTTCCACAAGAACCGATGAAAATCGTCGGCGACAGCGAGTCGACC
GGTACGCAGATCCACTTCAAGCCATCGGCTGAAACCTTCAAGAATATTCACTTCAGCTGGGACATCCTGG
CCAAGCGTATTCGTGAATTGTCCTTCCTCAACTCCGGTGTGGGTATCGTCCTCAAGGACGAACGCAGCGG
CAAGGAAGAGCTGTTCAAGTACGAAGGTGGTCTGCGTGCATTCGTTGAATACCTGAACACCAACAAGACT
GCGGTCAACCAGGTGTTCCACTTCAACATCCAGCGTGAAGACGGCATCGGCGTGGAAATCGCCCTGCAGT
GGAACGACAGTTTCAACGAGAACCTGTTGTGCTTCACCAACAACATTCCTCAGCGCGATGGCGGTACTCA
CTTGGTGGGTTTCCGTTCCGCACTGACGCGTAACCTGAACACCTACATCGAAGCCGAAGGCTTGGCCAAG
AAGCACAAAGTCGCCACCACCGGTGACGATGCCCGCGAAGGCCTGACCGCGATTATCTCGGTGAAAGTGC
CGGACCCTAAGTTCAGCTCCCAGACCAAAGACAAGCTGGTGTCTTCCGAGGTGAAGACCGCAGTGGAACA
GGAGATGGGCAAGTACTTCTCCGACTTCCTGCTGGAGAACCCGAACGAAGCCAAGCTGGTCGTCGGCAAG
ATGATCGACGCTGCACGTGCCCGTGAAGCTGCGCGTAAAGCCCGTGAGATGACCCGCCGTAAAGGCGCGC
TGGATATCGCTGGCCTGCCAGGCAAACTGGCTGACTGCCAGGAGAAGGACCCTGCCCTCTCCGAGCTGTA
TCTGGTGGAAGGTGACTCTGCTGGCGGTTCCGCCAAGCAGGGTCGTAACCGTCGCACCCAAGCCATCCTG
CCGTTGAAGGGTAAGATCCTCAACGTCGAGAAGGCCCGCTTCGACAAGATGATTTCTTCCCAGGAAGTTG
GCACCTTGATCACGGCGTTGGGCTGCGGTATGGCCGCGACGAGTACAACATCGACA
>gyrB_P.chlororaphis_SMMP3 (CP009290.1; JM49_30525)
AACGTCCCGGTATGTACATTGGTGACACCGACGATGGCAGCGGTCTGCACCATATGGTGTTCGAGGTGGT
CGATAACTCGATCGACGAAGCTCTGGCCGGCCACTGCGACGACATCAGCATCATCATCCATCCGGACGAA
TCCATTACCGTGCGTGACAACGGTCGCGGCATCCCGGTAGACGTGCATAAAGAAGAAGGCGTTTCCGCGG
CCGAGGTCATCATGACTGTGCTGCACGCCGGCGGTAAGTTCGACGACAACTCCTACAAAGTATCCGGCGG
TCTGCACGGTGTGGGTGTGTCGGTAGTGAACGCCCTGTCCGAAGAACTGGTCCTGACCGTTCGCCGCAGT
GGCAAGATCTGGGAACAGACATACGTTCACGGTGTGCCTCAGGCGCCTATGGCGATCGTCGGTGACAGCG
AAACCACCGGTACCCAGATTCACTTCAAGGCTTCTAGCGAGACCTTCAAGAACATCCATTTCAGCTGGGA
CATCCTGGCCAAGCGGATTCGTGAACTGTCCTTCCTCAACTCCGGTGTCGGTATCGTTCTGAAGGACGAG
CGCAGCGGCAAGGAAGAGCTGTTCAAGTACGAAGGCGGCCTGCGCGCATTCGTTGAATATCTGAACACCA
ACAAGACCGCGGTCAACCAGGTGTTCCACTTCAATGTGCAGCGTGAAGATGGCATCGGCGTGGAAATCGC
CCTGCAGTGGAACGACAGCTTCAACGAAAACCTGCAGTGCTTCACCAACAACATTCCGCAGCGCGACGGC
GGCACCCACCTGGTGGGCTTCCGTTCGGCACTGACGCGTAACCTGAACAACTACATCGAACAGGAAGGTC
TGGCGAAGAAGCACAAGGTCGCCACCACCGGTGACGATGCCCGCGAAGGCCTGACCGCGATCATTTCGGT
CAAGGTGCCGGATCCGAAGTTCAGCTCCCAGACCAAAGACAAGCTGGTGTCTTCCGAAGTGAAGACCGCA
GTCGAACAGGAAATGGGCAAGTACTTCTCCGACTTCCTGCTGGAAAACCCGAACGAAGCCAAGCTGGTGG

TCGGCAAGATGCTCGACGCCGCCCGTGCCCGTGAAGCGGCGCGTAAGGCTCGCGAGATGACCCGCCGTAA
AGGTGCGCTGGATATCGCCGGCCTGCCGGGCAAACTGGCGGACTGCCAGGAAAAAGACCCTGCCCTTTCC
GAACTCTACCTGGTGGAAGGTGACTCTGCTGGCGGCTCCGCCAAGCAGGGACGCAACCGTAAGACCCAGG
CGATTCTGCCGCTCAAGGGCAAGATCCTTAACGTCGAGAAAGCGCGTTTCGACAAGATGATTTCCTCGCA
AGAGGTCGGCACCTTGATCACTGCACTCGGTTGCGGCATCGGCCGTGAAGAGTACAACATCGACA
>gyrB_P.donghuensis_SVBP6 (NZ_JH650763.1; UW3_RS0111960)
CCGGTATGTACATTGGCGACACCGATGATGGTAGCGGCCTGCACCACATGGTGTTCGAGGTGGTCGATAA
CTCGATCGACGAGGCACTCGCCGGCCACTGCGATGACATCACCGTAATTATTCACCCGGACGAATCCATC
AGTGTTCGCGACAACGGTCGCGGCATTCCGGTTGACGTGCATAAAGAGGAAGGCGTTTCCGCCGCTGAGG
TCATCATGACCGTCCTCCACGCCGGCGGTAAATTCGATGACAACTCCTACAAGGTATCCGGCGGTCTGCA
CGGTGTAGGTGTGTCGGTGGTTAACGCCCTTTCCGAGCAGTTGATCCTCACCGTTCGCCGCAGTGGCAAG
ATCTGGGAACAGACCTACGTCCACGGTGTTCCACAAGCGCCGATGAAGATCGTTGGCGAAAGTGAAACCA
CCGGTACCCACATCCACTTCAAGCCGTCGGCTGAAACCTTCAAGAACATCCACTTCAGCTGGGACATCCT
GGCCAAGCGGATTCGTGAACTGTCCTTCCTCAACTCCGGCGTTGGCATCCTGCTGAAAGACGAGCGTTCG
GGCAAGGAAGAGTACTTCAAGTACGAAGGCGGTCTGCGTGCCTTCGTTGAGTACCTGAACACCAACAAGA
CGCCGGTCAACCAGGTGTTCCATTTCAATGTTCAGCGTGAAGACGGCGTGGGCGTGGAAATTGCCCTGCA
GTGGAACGACAGCTTCAACGAGAACCTGTTGTGCTTCACCAACAACATTCCGCAGCGCGACGGTGGTACC
CACCTGGTCGGTTTCCGCTCGGCGTTGACCCGTAACCTGAACAACTACATCGAGCAAGAAGGCCTGGCCA
AGAAGAACAAGGTTTCGACCACTGGCGACGACGCCCGTGAAGGTCTGACCGCGATCATCTCGGTGAAGGT
GCCGGATCCGAAGTTCAGCTCGCAGACCAAAGACAAGCTGGTGTCCTCGGAAGTGAAAACTGCCGTGGAA
CAGGAGATGGGCAAATACTTCTCCGACTTCCTGCTGGAGAACCCGAACGAAGCCAAGGCGGTTGTCGGCA
AGATGATCGACGCCGCTCGTGCCCGTGAAGCTGCACGTAAAGCGCGGGAAATGACTCGCCGCAAAGGTGC
GCTGGATATCGCCGGCCTGCCTGGCAAGCTGGCGGACTGCCAGGAGAAGGACCCTGCTCTTTCCGAACTG
TACCTGGTGGAGGGTGACTCCGCGGGTGGCTCGGCCAAGCAAGGCCGTAACCGCAAGACCCAGGCGATCC
TGCCGCTCAAGGGCAAGATCCTCAACGTCGAGAAAGCACGCTTCGACAAGATGATCTCGTCCCAGGAAGT
GGGCACCCTGATCACTGCGCTGGGCTGTGGTATCGGCCGCGAAGAGTACAACATCGACA
>gyrB_P.simiae_WCS417 (CP007637.1; PS417_00020)
ACGTCCCGGTATGTACATTGGCGACACTGATGACGGTAGCGGTCTGCACCACATGGTGTTCGAGGTGGTC
GACAACTCCATCGACGAAGCTCTGGCCGGTCACTGCGACGACATCAGCATCATCATCCACCCGGATGAGT
CTATTACCGTGCGCGACAATGGTCGCGGCATTCCGGTAGACGTACACAAAGAAGAAGGCGTTTCGGCAGC
AGAGGTCATCATGACCGTGCTCCACGCCGGCGGTAAGTTCGACGACAACTCCTATAAAGTTTCCGGCGGT
CTGCACGGTGTGGGCGTTTCGGTAGTGAACGCTCTTTCTGAAGAGTTGATCCTGACTGTTCGCCGCAGTG
GCAAGATCTGGGAACAGACCTACGTTCACGGTGTTCCAAAAGAACCGATGAAAATCGTCGGTGACAGTGA
AACCACCGGTACGCAGATCCACTTCAAGCCATCGGCTGAGACCTTCAAGAATATCCACTTTAGCTGGGAC
ATCCTGGCCAAGCGTATTCGTGAACTGTCCTTCCTGAACTCCGGTGTGGGTATCGTCCTCAAGGATGAGC
GCAGCGGTAAGGAAGAGCTGTTCAAGTATGAAGGTGGCCTGCGCGCATTCGTTGAATACCTGAACACCAA
CAAGACCGCGGTCAACCAGGTGTTCCACTTCAACATTCAGCGTGAAGACGGCATCGGCGTGGAAATCGCT
CTGCAGTGGAACGACAGCTTCAACGAGAACCTGTTGTGCTTCACCAACAACATTCCTCAGCGCGACGGTG
GTACTCACCTGGTGGGTTTCCGTTCCGCACTGACGCGTAACCTGAACACCTACATTGAAGCCGAAGGATT
GGCCAAAAAGCACAAAGTCGCCACCACCGGTGACGATGCGCGTGAAGGCCTGACCGCGATTATCTCGGTA
AAAGTACCTGATCCGAAGTTCAGCTCTCAGACCAAAGACAAGCTGGTGTCTTCCGAAGTGAAGACTGCGG
TGGAACAGGAGATGGGCAAGTATTTCTCCGACTTCCTGCTGGAGAACCCGAACGAAGCCAAGCTGGTTGT
CGGCAAGATGATCGATGCCGCGCGTGCCCGTGAAGCGGCGCGTAAAGCCCGTGAGATGACTCGTCGCAAA
GGCGCGCTGGATATCGCCGGCTTGCCAGGCAAACTGGCTGACTGCCAAGAGAAGGACCCTGCCCTCTCCG
AACTGTACCTGGTGGAAGGTGACTCTGCTGGCGGTTCCGCCAAGCAGGGTCGTAACCGTCGCACCCAAGC
CATCCTGCCGTTGAAAGGTAAGATCCTCAACGTCGAGAAGGCTCGCTTCGACAAGATGATTTCCTCCCAG
GAAGTCGGCACCTTGATCACGGCGTTGGGCTGCGGTATCGGCCGCGACGAGTACAACATCGACAAGTTGC
GCTATCACAACATCATCATCATGACC

Genbank accession numbers:

gyrB of *P. sihuiensis* 2013: MG757236
gyrB of *P. stutzeri* 2014: MG757237
gyrB of *P. stutzeri* 2018: MG757238
gyrB of *Pseudomonas* sp. CF5: MG757239
gyrB of *P. sihuiensis* N23: MG757240
gyrB of *Pseudomonas* sp. 1008: G757243
gyrB of *P. chlororaphis* SMMP3: G757241
gyrB of *P. donghuensis* SVBP6: MG757242

Supplementary Figure S1. Gradient PCR of *gyrB* gene. The reactions were performed using genomic DNA from *P. protegens* Pf-5 or *P. protegens* CHA0 lysate as template. Reactions were carried out with primers listed in Table 2 with the annealing temperatures shown above each lane. The negative control without DNA (C-) was assayed at 49 °C. Molecular mass marker (M) correspond to the 100-bp DNA ladder (Embiotec, Argentina), and the 500 bp band is marked with a white arrow. Specificity was obtained at 57 °C, where the double band dissapears using both kind of templates.

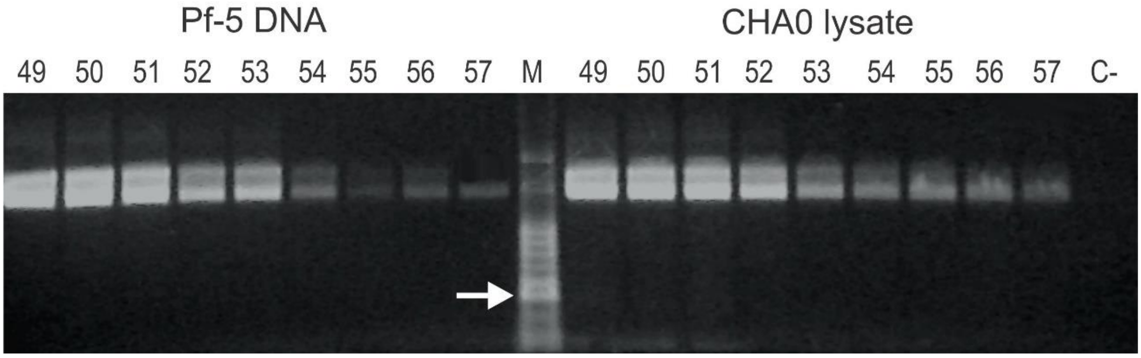

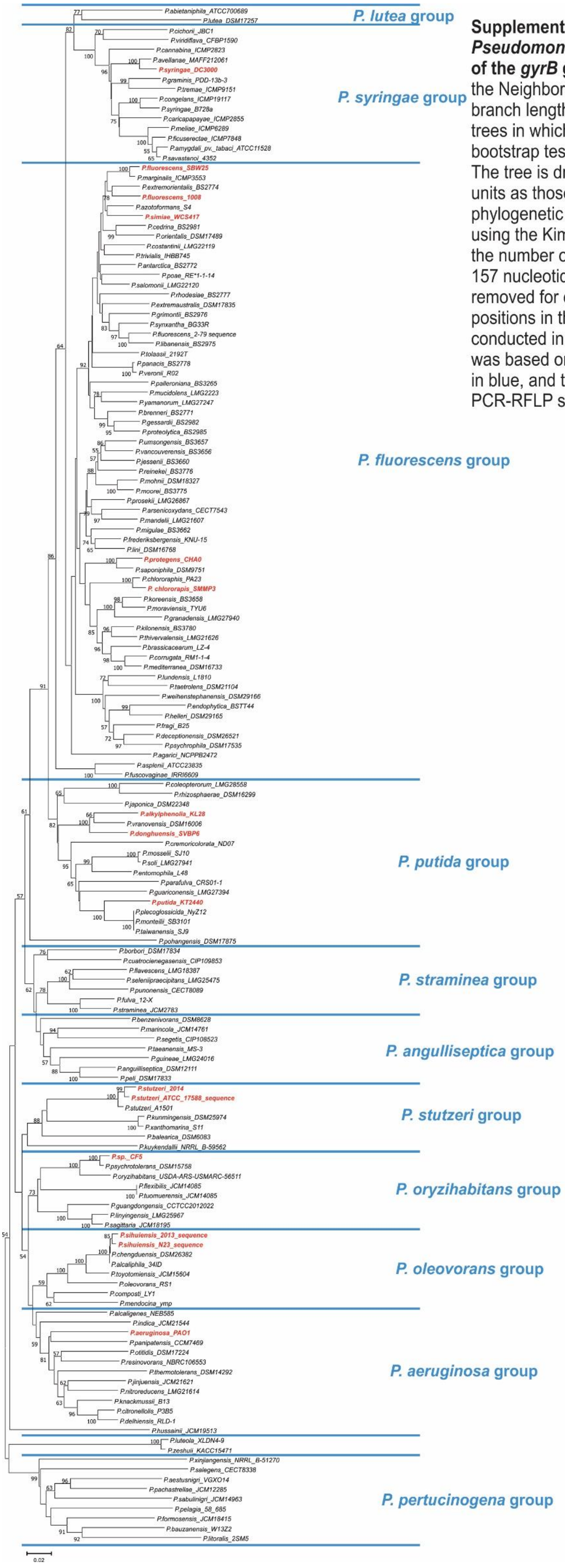

Supplementary Figure S2. **Evolutionary relationships of *Pseudomonas* species based on the amplicon sequences of the *gyrB* gene**. The evolutionary history was inferred using the Neighbor-Joining method. The optimal tree with the sum of branch length = 7.21 is shown. The percentage of replicate trees in which the associated taxa clustered together in the bootstrap test (1000 replicates) are shown next to the branches. The tree is drawn to scale, with branch lengths in the same units as those of the evolutionary distances used to infer the phylogenetic tree. The evolutionary distances were computed using the Kimura 2-parameter method and are in the units of the number of base substitutions per site. The analysis involved 157 nucleotide sequences. All ambiguous positions were removed for each sequence pair. There were a total of 1476 positions in the final dataset. Evolutionary analyses were conducted in MEGA7. *Pseudomonas* groups classification was based on Gomila et al (2015). Groups are indicated in blue, and the isolates employed in sequencing and PCR-RFLP set up assays are shown in red.

Supplementary Figure S3. Polymerase chain reaction targetting the *gyrB* gene of non-pseudomonads strains of our laboratory collection, with the newly designed primers described in this study. We employed thermal lysates as templates for the PCR reaction described in Material and Methods section, with primers from in the Table 2. Strains were as follows: 1, *Shinorhizobium meliloti* 2011; 2, *Rhizobium etlii* CE3; 3, *Rhizobium fredii* NGR234; 5, *Escherichia coli* K12; 6, *Chromobacterium violaceum* ViR07; 7, *Azospirillum brasilense* ATCC29710; 8, *Serratia marcecens* AS-1; 9, *Burkholderia cepacia* (environmental isolate); 10, *Salmonella* spp.; 11, *Staphilococcus aureus* ATCC 25923; *Agrobacterium tumefaciens* NTL4; 13, *Bacillus cereus* (environmental isolate); 14; *Bacillus subtilis* (environmental isolate);15, *Vibrio harveyii* BB170; 16, *Pantoea* spp. (environmental isolate); 17, negative control (without DNA) ; 18, positive control (*P. protegens* CHA0 as template). M, molecular weigth marker λBstEII (Embiotec, Buenos Aires, Argentina). A DNA band of 1371 bp is indicated with an arrow.

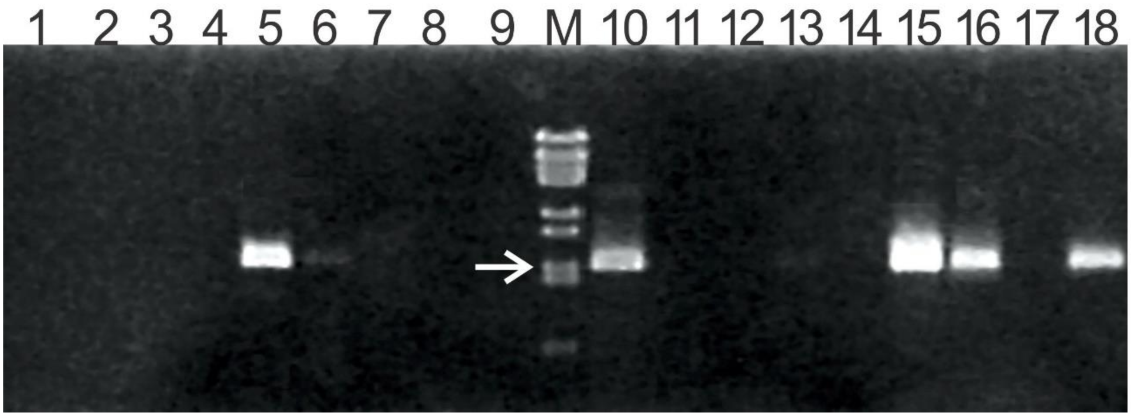

Supplementary Table S1. Sequences of *gyrB* gene from the *Pseudomonas* species included in the analysis

***Pseudomonas* species [a]**	**Strain**	**GenBank accession number**	**Observations**
P. aeruginosa	PA01	AE004091.2	
P. abietaniphila	ATCC 700689	FNCO01000018.1	
P. aestusnigri	VGXO14	NZ_NBYK01000006.1	
P. agarici	NCPPB2472	CP014135.1	
P. alcaliphila	34	ANGB01000004.1	
P. alcaligenes	NEB 585	CP014784.1	
P. alkylphenolia	KL28	CP009048.1	
P. amygdali pv. tabaci	ATCC 11528	LCWS01000009.1	
P. anguilliseptica	DSM 12111	NZ_FNSC01000001.1	
P. antarctica	BS2772	LT629704.1	
*P. argentinensis**	LMG 22563	FN554170.1	*gyrB* sequence not complete
P. arsenicoxydans	CECT 7543	LT629705.1	
P. asplenii	ATCC 23835	LT629777.1	
*P. asturiensis**	LPPA 221	HF546083.1	*gyrB* sequence not complete
*P. asuensis**	CP155-2	--	*gyrB* sequence not available
P. avellanae	MAFF 212061	NKQU01000008.1	
*P. azotifigens**	JCM 12708	KM103930.1	*gyrB* sequence not complete
P. azotoformans	S4	CP014546.1	
*P. baetica**	CECT 7720	HE800470.1	*gyrB* sequence not complete
P. balearica	DSM 6083	CP007511.1	
P. bauzanensis	W13Z2	JFHS01000003.1	
P. benzenivorans	DSM 8628	NZ_FNCT01000008.1	
P. borbiri	DSM 17834	NZ_FOWX01000035.1	
P. brassicacearum	LZ-4	JNCR01000040.1	
P. brenneri	BS2771	NZ_LT629800.1	
*P. caeni**	CECT 7778	HE800473.1	*gyrB* sequence not complete
P. cannabina	ICMP 2823	LJPX01000020.1	
*P. carboxydohydrogena**	DSM 1083	--	*gyrB* sequence not available
P. caricapapayae	ICMP2855	LJPW01000146.1	
P. cedrina	BS2981	LT629753.1	
P. chengduensis	DSM 26382	NZ_FMZQ01000001.1	
P. chlororaphis	PA23	CP008696.1	
P. cichorii	JBC1	CP007039.1	
P. citronellolis	P3B5	CP014158.1	
P. coleopterorum	LMG 28558	NZ_FNTZ01000001.1	
P. composti	LY1	LSSW01000029.1	
P. congelans	ICMP19117	LJQB01000051.1	
P. corrugata	RM1-1-4	CP014262.1	
P. costantinii	LMG 22119	FN554180.1	
P. cremoricolorata	ND07	CP009455.1	
P. cuatrocienegasensis	CIP 109853	NZ_FOFP01000011.1	
P. deceptionensis	DSM 26521	JYKX01000009.1	
P. delhiensis	RLD-1	FZPC01000017.1	
P. donghuensis	SVBP6	SUB3052350	
*P. duriflava**	KCTC 22129	HE800478.1	

P. endophytica	BSTT44	LLWH01000045.1	
P. entomophila	L48	CT573326.1	
P. extremaustralis	DSM 17835	LT629689.1	
P. extremorientalis	BS2774	LT629708.1	
P. ficuserectae	ICMP7848	LJQJ01000012.1	
P. flavescens	LMG 18387	NZ_FNDG01000003.1	
P. flexibilis	JCM 14085	JTAK01000006.1	
P. fluorescens	SBW25	AM181176.4	
P. formosensis	JCM18415	NZ_FOYD01000007.1	
P. fragi	B25	NZ_JH604624.1	
P. frederiksbergensis	KNU-15	CP023466.1	
P. fulva	12-X	CP002727.1	
P. fuscovaginae	IRRI6609	JSYZ01000014.1	
*P. glareae**	NR_145562.1	--	*gyrB* sequence not available
P. gessardii	BS2982	NZ_FNKR01000003.1	
P. graminis	PDD-13b-3	MTSB01000006.1	
P. granadensis	LMG 27940	LT629778.1	
P. grimontii	BS2976	NZ_FNKM01000002.1	
P. guangdongensis	CCTCC 2012022	LT629780.1	
P. guariconensis	LMG 27394	NZ_FMYX01000004.1	
P. guineae	LMG 24016	NZ_FOQL01000001.1	
P. helleri	DSM 29165	JYLD01000021.1	
*P. helmanticensis**	CECT 8548	LT601023.1	*gyrB* sequence not complete
P. hussainii	JCM 19513	NZ_FOAS01000013.1	
P. indica	JCM 21544	FNFD01000025.1	
P. japonica	DSM 22348	NZ_BBIR01000012.1	
P. jessenii	BS3660	FNTC01000002.1	
P. jinjuensis	JCM 21621	FNIJ01000013.1	
P. kilonensis	BS3780	FNTT01000002.1	
P. knackmussii	B13	HG322950.1	
P. koreensis	BS3658	LT629687.1	
P. kunmingensis	DSM 25974	NZ_FORS01000009.1	
P. kuykendallii	NRRL B-59562	FNNU01000007.1	
P. libanensis	BS2975	LT629699.1	
P. lini	DSM 16768	JYLB01000011.1	
P. linyingensis	LMG 25967	FNZE01000025.1	
P. litoralis	2SM5	LT629748.1	
P. lundensis	L1810	NQKH01000001.1	
*P. lurida**	DSM 15835	JN589908.1	*gyrB* sequence not complete
P. lutea	DSM 17257	JRMB01000002.1	
P. luteola	XLDN4-9	NZ_ALAT01000183.1	
P. mandelii	LMG 21607	LT629796.1	
P. marginalis	ICMP 3553	LKEG01000034.1	
P. marincola	JCM 14761	NZ_FPBC01000007.1	
*P. matsuisoli**	CC-MHH0089	KM249139.1	*gyrB* sequence not complete
P. mediterranea	DSM 16733	LT629790.1	
P. meliae	ICMP6289	LJQT01000046.1	
P. mendocina	ymp	CP000680.1	
*P. meridiana**	CIP 108465	FN554203.1	*gyrB* sequence not complete

P. migulae	BS3662	NZ_FNTY01000002	
P. mohnii	DSM 18327	NZ_FNRV01000001.1	
P. monteilii	SB3101	CP006979.1	
P. moorei	BS3775	FNKJ01000003.1	
P. moravinesis	TYU6	NRST01000001.1	
P. mosselii	SJ10	CP009365.1	
P. mucidolens	LMG 2223	LT629802.1	
*P. nitritireducens**	LMG 25966	LT601026.1	*gyrB* sequence not complete
P. nitroreducens	LMG 21614	FZOM01000003.1	
*P. oceani**	DSM 100277	KU187947.1	*gyrB* sequence not complete
P. oleovorans	RS1	FNZC01000014.1	
P. orientalis	DSM 17489	JYLM01000012.1	
P. oryzihabitans	56511	CP013987.1	
P. otitidis	DSM 17224	FOJP01000007.1	
P. pachastrellae	JCM 12285	NZ_FOUD01000003.1	
P. palleroniana	BS3265	NZ_FNUA01000002.1	
P. panacis	BS2778	NZ_FNSP01000004.1	
P. panipatensis	CCM7469	FNDS01000012.1	
P. parafulva	CRS01-1	CP009747.1	
P. pelagia	58 685	NWMT01000063.1	
P. peli	DSM 17833	NZ_FMTL01000003.1	
*P. pertucinogena**	JCM 11950	DQ350613.2	*gyrB* sequence not complete
P. plecoglossicida	NyZ12	CP010359.1	
P. poae	RE*1-1-14	CP004045.1	
P. pohangensis	DSM 17875	LT629785.1	
*P. populi**	KBL-4-9	KJ561381.1	*gyrB* sequence not complete
P. prosekii	LMG 26867	LT629762.1	
P. protegens	CHA0	CP003190.1	
P. proteolytica	BS2985	NZ_FNTR01000004.1	
P. psychrophila	DSM 17535	JYKZ01000013.1	
P. psychrotolerans	DSM 15758	FMWB01000002.1	
P. punonensis	CECT 8089	FRBQ01000001.1	
P. putida	KT2440	AE015451.2	
P. reinekei	BS3776	LT629709.1	
P. resinovorans	NBRC 106553	AP013068.1	
P. rhizosphaerae	DSM 16299	CP009533.1	
P. rhodesiae	BS2777	LT629801.1	
P. sabulinigri	JCM 14963	LT629763.1	
P. sagittaria	JCM 18195	NZ_FOXM01000021.1	
P. salegens	CECT 8338	LT629787.1	
*P. salina**	XCD-X85	KP098570.1	*gyrB* sequence not complete
P. salomonii	LMG 22120	MDFI01000010.1	
P. saponiphila	DSM 9751	NZ_FNTJ01000002.1	
P. savastanoi	4352	LGKR01000057.1	
P. segetis	CIP 108523	NZ_FZOG01000003.1	
P. seleniipraecipitans	LMG 25475	FNBM01000012.1	
P. simiae	WCS417	CP007637.1	
P. soli	LMG 27941	NZ_FOEQ01000003.1	
P. straminea	JCM 2783	NZ_FOMO01000001.1	

P. stutzeri	A1501	CP000304.1	
P. synxantha	BG33R	CM001514.1	
P. syringae pv. syringae	B728a	CP000075.1	
P. taeanensis	MS-3	AWSQ01000001.1	
P. taetrolens	DSM 21104	JYLA01000004.1	
P. taiwanensis	SJ9	AXUP01000002.1	
P. thermotolerans	DSM 14292	AQPA01000058.1	
P. thivervalensis	LMG 21626	LRSO01000016.1	
P. tolaasii	NCPPB 2192	CP020369.1	
P. toyotomiensis	JCM 15604	FOXK01000005.1	
P. tremae	ICMP9151	LJRO01000492.1	
P. trivialis	IHBB745	CP011507.1	
P. tuomuerensis	JCM 14085	KR706346.1	
*P. turukhanskensis**	IB-1.1	LT219440.1	*gyrB* sequence not complete
P. umsongensis	DSM 16611	NIWU01000003.1	
P. vancouverensis	BS3656	LT629803.1	
P. veronii	R02	CP018420.1	
P. viridiflava	CFBP 1590	LT855380.1	
P. vranovensis	DSM 16006	AUED01000002.1	
P. weihenstephanensis	DSM 29166	JYLF01000003.1	
P. xanthomarina	S11	CCYE01000001.1	
*P. xiamenensis**	DSM 22326	DQ350612.2	*gyrB* sequence not complete
P. xinjiangensis	NRRL B-51270	LT629736.1	
P. yamanorum	LMG 27247	NZ_LT629793.1	
P. zeshuii	KACC 15471	FQYS01000008.1	
*P. zhaodongensis**	NEAU-ST5-21	KP151495.1	*gyrB* sequence not complete

[a] The analysis included all the *gyrB* sequences available on Genbank at date (September 2017), based on the species list described in LPSN.

* Sequences with an asterisk were not included because their complete *gyrB* sequence were not available on Genbank

Supplementary Table S2. Coverage and accuracy of the sequencing of *gyrB* gene from the *Pseudomonas* strains tested *in vitro*

**Strain designation**	**Amplicon size [a]**	**Sequence obtained**	**Coverage**	**Accuracy [e]**
P. aeruginosa PAO1	1467 bp	1376 bp	93.8 %	
P. alkylphenolica KL28	1464 bp	1389 bp	94.9 %	
P. chlororaphis SMMP3	1464 bp	1395 bp	95.3 %	
P. donghuensis SVBP6	1464 bp	1389 bp	94.9 %	
Pseudomonas sp. 1008	1464 bp	1397 bp	95.4 %	
P. fluorescens 2-79	1464 bp	1391 bp	95.0 %	
P. fluorescens SBW25	1464 bp	1386 bp	94.7 %	
P. protegens CHA0	1464 bp	1400 bp	95.6 %	
P. putida KT2440	1467 bp	1399 bp	95.4 %	
P. sihuiensis 2013 [b]	1467 bp	1387 bp	94.5 %	99.1 % *P. sihuiensis* KCTC 32246
P. sihuiensis N23 [b]	1467 bp	1402 bp	95.6 %	99.5 % *P. sihuiensis* KCTC 32246
P. simiae WCS417	1464 bp	1426 bp	97.4 %	
P. stutzeri 2014 [c]	1461 bp	1380 bp	94.5 %	98.2 % *P. stutzeri* DSM 4166
P. stutzeri 2018 [c]	1461 bp	1379 bp	94.4 %	99.4 % *P. stutzeri* DSM 4166
P. stutzeri ATCC 17588	1461 bp	1373 bp	94.0 %	
P. syringae pv. *tomato* DC3000	1464 bp	1397 bp	95.4 %	
Pseudomonas sp. CF5 [d]	1467 bp	1383 bp	94.3 %	99.8% *P. psychrotolerans* L19

[a] Fragment size calculated in silico with the AmplifX software v.1.7.0, from the *gyrB* complete sequence of the corresponding strain.
[b] Amplified fragment size based on the *P. sihuiensis* KCTC 32246 *gyrB* gene.
[c] Amplified fragment size based on the *P. stutzeri* ATCC 17588 *gyrB* gene.
[d] Amplified fragment size based on the *P. psychrotolerans* L19 *gyrB* gene.
[e] Percentage values of accuracy comparing with the corresponding type strain sequences from BlastN analyses. Type strains showed 100% accuracy with their corresponding sequences on Genbank database
